# Dynamic evolution of the active center driven by hemilabile coordination in Cu/CeO_2_ single-atom catalyst

**DOI:** 10.1038/s41467-023-38307-w

**Published:** 2023-05-02

**Authors:** Zheng Chen, Zhangyun Liu, Xin Xu

**Affiliations:** 1 Collaborative Innovation Center of Chemistry for Energy Materials, Shanghai Key Laboratory of Molecular Catalysis and Innovative Materials, MOE Key Laboratory of Computational Physical Sciences, Department of Chemistry, Fudan University, Shanghai, 200433 P. R. China; 2 Hefei National Laboratory, Hefei, 230088 P. R. China

**Keywords:** Heterogeneous catalysis, Reaction kinetics and dynamics, Theory and computation, Materials for energy and catalysis

## Abstract

Hemilability is an important concept in homogeneous catalysis where both the reactant activation and the product formation can occur simultaneously through a reversible opening and closing of the metal-ligand coordination sphere. However, this effect has rarely been discussed in heterogeneous catalysis. Here, by employing a theoretical study on CO oxidation over substituted Cu_1_/CeO_2_ single atom catalysts, we show that dynamic evolution of metal-support coordination can significantly change the electronic structure of the active center. The evolution of the active center is shown to either strengthen or weaken the metal-adsorbate bonding as the reaction proceeds from reactants, through intermediates, to products. As a result, the activity of the catalyst can be increased. We explain our observations by extending hemilability effects to single atom heterogenous catalysts and anticipate that introducing this concept can offer a new insight into the important role active site dynamics have in catalysis toward the rational design of more sophisticated single atom catalyst materials.

## Abstract (web summary)

This work explores the concept of hemilability in single atom catalysts. The results imply that this effect can promote reactant activation and product desorption simultaneously to provide a potential approach to circumvent the Sabatier volcano.

## Introduction

There is a continuous demand for new strategies of catalyst design based on the fundamental understanding^1–3^, as our ultimate goal is to develop more active, more selective, and less expensive catalysts. This actually holds the key to eventually solve the energy problems and the environmental problems for a sustainable development of our society. For heterogeneous catalysis, the well-known Sabatier principle states that an optimal catalyst should bind the adsorbates neither too weakly to activate the reactants, nor too strongly to desorb the products. On top of this principle, theoretical approaches have been devloped to identify the top of the activity volcanoes for rational catalyst design, using the scaling relationship among adsorbates on a familily of static catalysts with structurally well-defined active centers^1,2,4–8^. Recently, the use of the scaling relationship has also been extended to the design of homogeneous catalysts^9,10^, although the common strategies to improve the catalytic activity and selectivity in homogeneous catalysis are to manipulate the active metal center and its local coordination environments^11,12^.

Single-atom catalysts (SACs), which contain isolated metal atoms singly dispersed on the supports, are highly attractive due to their high atom economy and distinctive performances for a wide variety of chemical reactions^13–20^. The success of SACs largely relies on the well-defined bonding of isolated atoms with the support. On the other hand, SACs can combine the advantages of both heterogeneous and homogeneous catalysts, whose design strategy can rely not only on the scaling relationship on well-defined active centers^21–23^, but also on tailoring the local coordination environments^23–26^. Although it has now been emphasized that the local coordination environments of SACs can dynamically change in response to the change of reaction conditions, such that the initial SAC evolved to different structures under^27–30^, e.g., reduction and oxidation reaction conditions, respectively^27,28^, little attention has been paid to the fact that even when the steady state under a given reaction condition has been reached, the coordination environments between the metal center and the adsorbates are still dynamically changing, as the adsorbates change from the reactants to the intermediates, and eventually to the products, as the reaction proceeds during a catalytic cycle. The dynamic metal-adsorbate coordination can compete with the metal-support coordination, as exemplified by a Pd SAC, where the flexible host material provided an adaptive coordination environment to facilitate each catalytic step^31^. Therefore, an advanced design strategy for SACs should consider this dynamic metal-support coordination along with a dynamic metal-adsorbate coordination.

**Fig. 1:**
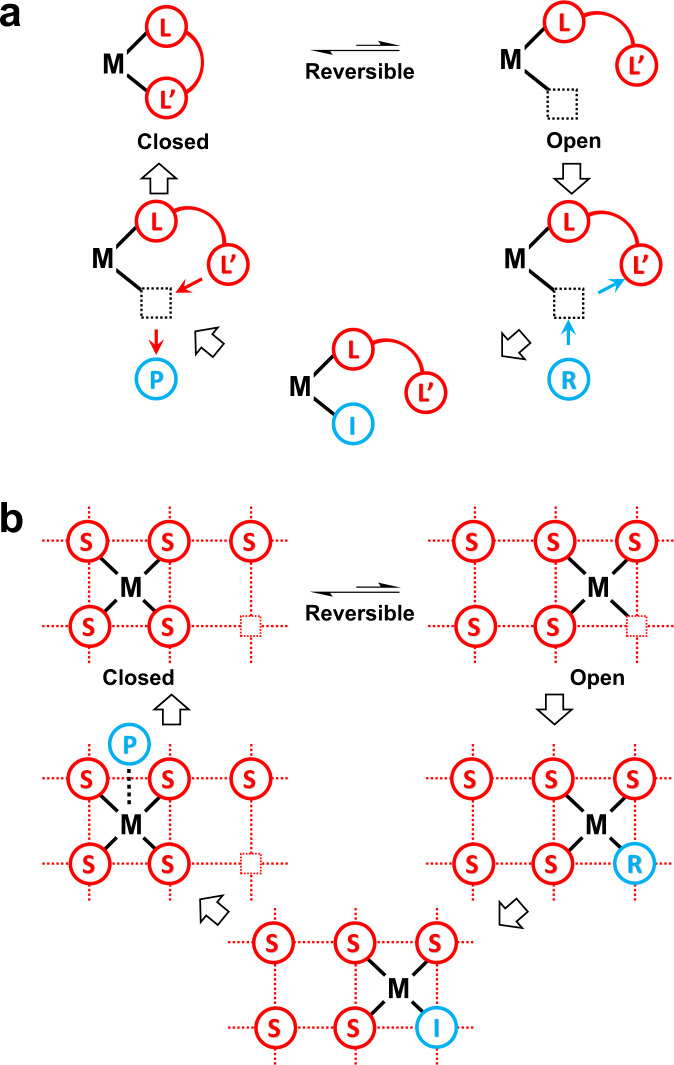
Schemes of Hemilability. **a** Hemilability in homogeneous catalysis. A hemilabile ligand (L-L’) has a weak chelating group (L’) that is capable of occupying and releasing reversibly the coordination site at the reactive metal center (M). In the presence of the reactant (R), the hemilabile ligand is in the open state to facilitate the formation of the M-R coordination, which is then transformed to the M-intermediate (M-I) coordination along the reaction pathway. Upon the product (P) formation, the hemilabile ligand returns to the closed state, where the M-L’ coordination wins over the M-P coordination to facilitate the release of the product. **b** A possible mechanism which may be viewed as hemilability in heterogeneous catalysis. There are defect sites and standby sites on the support, where the metal center can migrate from a full metal-support coordination site (the closed state) to a defect site (the open state). The activation of the reactant (R) is facilitated in the open state, while the release of the product (P) is facilitated when the active center is transformed back to the closed state.

Hemilability is an important and useful concept in homogeneous catalysis^32–37^. As shown in Fig. 1a, hemilability is featured as a reversible opening and closing of a coordination site, where the labile metal-ligand coordination is in the “open” state, displaced by the metal-reactant/intermediate coordination, while in the “closed” state, displacing the metal-product coordination^32^. The significance of the hemilabile ligand is emphasized in homogeneous catalysis as one of the effective ways to tune the reactivity of the catalyst, where the open state favors the reactant activation, while the closed state favors the product elimination^33^. However, hemilability has rarely been discussed in heterogeneous catalysis such as in SAC, which, we envision, is highly possible to exist and function due to the competition between the metal-support coordination and the metal-adsorbate coordination. Naturally, unlike that in homogeneous catalysts, there are defect sites and standby sites on the support, where the metal center can migrate to an appropriate position to optimize the coordinations for the metal-support and the metal-adsorbate interactions during the reaction. Therefore, the reversible opening and closing of a hemilabile metal-support coordination in SACs shall be similar to, but not all the same as that in homogeneous catalysts. Figure 1b illustrates a possible mechanism for a dynamic metal-support coordination along the reaction pathway when the adsorbate changes from the reactant to the intermediate, and eventually to the product, as the reaction proceeds during a catalytic cycle, which may be viewed as hemilability in heterogeneous catalysis.

CO oxidation is the most widely used prototypical model reaction, which reveals the richness and beauty of heterogeneous catalysis^38^. In fact, the concept of SAC was firstly demonstrated by CO oxidation on Pt_1_/FeO_x_^13^, while the phonon-assisted dynamics for the charges and oxidation states in SAC was firstly displayed by CO oxidation on Pt_1_/CeO_2_^39^. The corresponding mechanism for the CO oxidation on SACs has been extensively investigated^13,39–42^. Here we noticed that a sintered Cu/CeO_2_ was reported^42^, which showed a very high reactivity in catalyzing the CO oxidation, overshooting that of other reported copper catalysts by many times. Although an atomically dispersed Cu on ceria was identified by the experimental characterization, why this sintered Cu/CeO_2_ SAC showed such a high CO oxidation reactivity remains elusive^42^.

Herein, we employed density functional theory (DFT) calculations to study the CO oxidation on the substituted Cu_1_/CeO_2_ catalyst. The results unearth the existence of a dynamic coordination of the Cu ion, which plays a key role in achieving the observed high activity of CO oxidation with this substituted Cu_1_/CeO_2_ catalyst. We relate our observation to hemilability. While the linear scaling relationships impose the dependences of the adsorption energies of different intermediates on a structurally well-defined active center in a static catalyst, hemilability with the reversible opening and closing of the metal-support coordination allows for both favorable reactant activation and product desorption simultaneously by tuning the structure of the active center dynamically. In fact, the opening and closing of this hemilabile coordination was found here to significantly change the d-electron structure of the Cu ion, which helped to intensify or weaken the appropriate metal-adsorbate bonding. The importance of this dynamic coordination was further illustrated by a direct comparison with other models of SACs for their less activity in CO oxidation due to either a too flexible or a too rigid metal-support coordination, respectively, as compared to the substituted Cu_1_/CeO_2_ catalyst with hemilability. Therefore, we expect that introducing the concept of hemilability may offer a useful design strategy for SACs to circumvent the static optimum of the Sabatier volcano^2,25,43,44^.

## Results and discussion

### Hemilability in CO oxidation mechanism

To model the CO oxidation on the substituted Cu_1_/CeO_2_ catalyst, the Perdew–Burke–Ernzerhof functional^45^ together with the Hubbard U correction^46^, i.e., PBE + U, was employed. Potential energy surfaces and the corresponding structure changes were obtained by DFT calculations, where the transition state theory was utilized to describe the dynamic metal-support coordinations. Then, kinetic Monte Carlo (KMC) simulations were employed to calculate the turnover frequency (TOF), reaction orders of CO and O_2_, and apparent activation energy to make a direct comparison with the experiment^42^. The valence of the Cu ion in SAC was estimated by comparing the Bader charge^47,48^ to those in the Cu^(I)^_2_O and Cu^(II)^O bulks (Supplementary Table 1). A Cu-substituted CeO_2_(111) surface model was used, since the (111) planes were often the preferentially exposed facets when ceria was treated at high temperature^49,50^. The generation of an oxygen vacancy (V_O_) was found to be spontaneous^51^, as one pair of Ce^4+^−O^2−^ on the (111) surface was replaced by a pair of Cu^2+^−V_O_ to maintain the charge balance. After optimization, the Cu ion possessed a square planar configuration for the substituted Cu_1_/CeO_2_ model catalyst, bonding to four lattice oxygens (Supplementary Fig. 1a). The stability of the substituted Cu_1_/CeO_2_(111) model under reaction conditions was also examined (Supplementary Fig. 2). More details for the computational methods and the models used can be found in the Supplementary Methods 1.1–1.7, Supplementary Figs. 1–3 and Supplementary Tables 1–3.

**Fig. 2:**
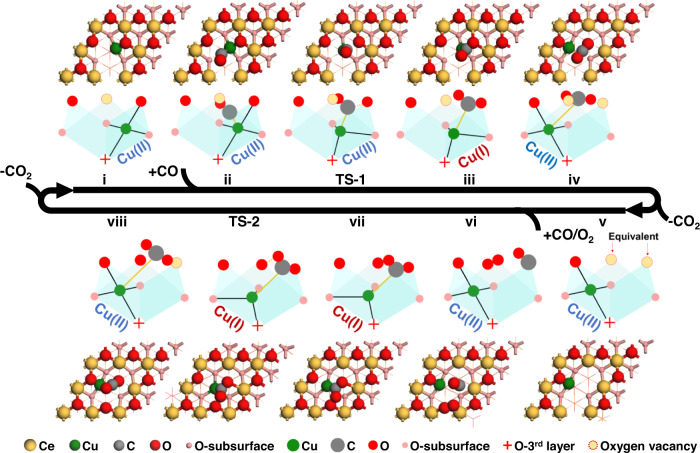
Optimized structures for Cu_1_/CeO_2_(111) in the catalytic cycle with the hemilabile metal-support coordination. In the first half of the catalytic cycle (i–iv), CO is oxidized by consuming a lattice oxygen. In the second half of the catalytic cycle (v–viii), CO is oxidized by a co-adsorbed oxygen molecule, which, at the same time, results in the lattice oxygen regeneration to complete the catalytic cycle. The sidewiew cartoon scheme represents the coordination environments of the Cu ion. The light yellow circles indicate the locations of the oxygen vacancies. The positions for the two oxygen vacancies in state v are equivalent, such that the catalyst is regenerated after CO_2_ desorption from state viii. The metal-support coordinations change in company with the changes of the valence of Cu, i.e., open for Cu(I) and closed for Cu(II), while the valence of Cu in different states are estimated by comparing the Bader charges to those in bulk Cu^(II)^O and Cu_2_^(I)^O.

Figure 2 summarizes the optimized structures of intermediates and transition states in the catalytic cycle on Cu_1_/CeO_2_(111), along with the corresponding scheme for the local coordination environment of the Cu ion in company with the corresponding valence of Cu. In the first half of the cycle (i–iv in Fig. 2), the adsorbed CO (i.e., CO*) on the spontaneously formed V_O_ consumes a nearby lattice oxygen, which creates a new V_O_. In the second half of the catalytic cycle (v–viii in Fig. 2), an associative CO + O_2_ pathway occurs, where CO and O_2_ co-adsorb to firstly occupy these two V_O_ sites, and then proceed with the subsequent CO oxidation and a lattice oxygen regeneration. After CO_2_ desorption from state viii, the catalytic cycle is complete and the catalyst is regenerated. Other pathways have also been attempted, which turned out to be less favorable on Cu_1_/CeO_2_(111) (Supplementary Fig. 3). Based on the current mechanism, KMC simulations have been carried out and the results are presented in Supplementary Method 1.6 and Supplementary Fig. 4, which provides a good comparison with the experimental results^42^.

As shown in Fig. 2, one of the four Cu-O coordination sites is opened and displaced by a metal-adsorbate coordination Cu-CO_2_* (state iii) with a lattice O being consumed to form a bent CO_2_* after surmounting TS-1. It is important to notice that, in state iv, the metal-support coordination site is closed by Cu migrating to another equivalent square and bonding to four lattice oxygens. Simultaneously, the metal-adsorbate coordination is weakened, as demonstrated by the observation that an active bended CO_2_*, as in state iii and TS-1, is transformed to an inactive linear CO_2_* as in state iv (see Supplementary Fig. 5 for more details of structure changes from state iii to state iv). In the second half of the catalytic cycle, DFT optimizations lead to the co-adsorption of CO* and O_2_*, which can only exist on two adjacent V_O_ sites as shown in state vi. The formation of the active OCOO* species is barrierless, forming a Cu-C adsorbate coordination spontaneously with an opening of the Cu-O support coordination site as shown in state vii. After surmounting TS-2 to form the second CO_2_, the re-coordination (the closing) of the Cu-O is achieved simultaneously as shown in state viii, while a lattice oxygen is regenerated as shown in state viii (see Supplementary Fig. 6 for more details of structure changes from state vii to state viii). To view the dynamic metal-support coordination more intuitively, an ab initio molecular dynamic (AIMD) simulation is also performed on the OCOO* dissociation (Supplementary Fig. 7 and Supplementary Movies 1–2). The simulation starts from state vii, where the structure with the opening of the Cu-O support coordination site is seen to be filled in by the Cu coordination with the OCOO* adsorbate, while the simulation ends at state viii, where the re-coordination of the Cu-O and the OCOO* dissociation is seen to happen simultaneously. This picture of dynamic metal-support coordination is also indicated in the images from the calculations using the nudged elastic band method^52^ (Supplementary Fig. 8). Therefore, the reversible opening and closing of the Cu-O metal-support coordination site clearly demonstrates a diversiform hemilabile behavior in SACs as compared to that in homogeneous catalysis, which is achieved here in company with the migration of the active Cu ion to the nearby position and the consumption/regeneration of the lattice oxygen.

**Fig. 3:**
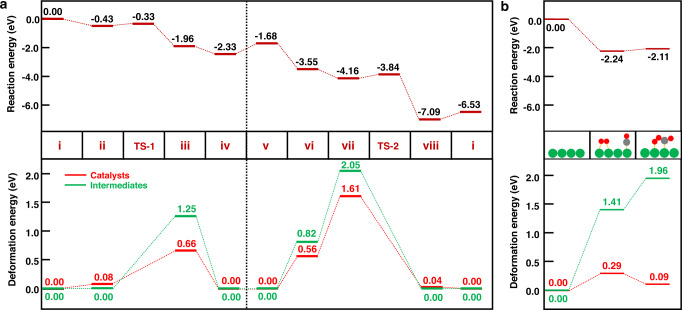
The reaction energy landscape and the decomposed deformation energy of each state. For (**a**) CO oxidation on the substituted Cu_1_/CeO_2_, and for (**b**) OCOO* formation on the Cu(111) surface. The deformation energy is defined as the energy difference between the energies in the conformation confined in the combined system and that in the optimal configuration in the free state of the individual component (see details in Supplementary Method 1.3). As shown in Fig. 3a (bottom), the deformation energies are large for both the adsorbates (green line) and the catalysts (red line) for states with the metal-support coordination site being opened (state iii and state vii), which intensify the strong metal-intermediate interaction to stabilize the intermediates. On the contrary, the deformation energies of the catalysts are small for states with the metal-support coordination site being closed (state iv and state viii), where the metal-adsorbate interactions are weakened so as to facilitate the desorption of the product. Figure 3b (bottom) shows that the deformation energies of an extended Cu(111) surface (red line) are much mild, although the deformation energies are still large for the adsorbates (green line).

Figure 3a (top) shows the reaction landscape in terms of electronic energy. As is clear, the CO oxidation on the substituted Cu_1_/CeO_2_(111) surface goes very smoothly, where the highest barrier appears at the OCOO* dissociation step (from state vii to state viii) as 0.32 eV only. Note that, although CO_2_* desorption step seems to be more endothermic in electronic energy, it is actually a fast process due to its larger pre-exponential factor than that of surface reactions (see Supplementary Fig. 9 for the free energy landscape at 393.15 K).

To understand the role that the dynamic coordination plays in controlling the reactivity of the substituted Cu_1_/CeO_2_(111), we have analyzed the reaction energy landscape, the deformation energies (see details in Supplementary Method 1.3) for the components of the adsorbate and the catalyst, and the Cu-adsorbate bond strength (see Supplementary Table 4), respectively, in each state. As shown in Fig. 3a (bottom), for states with the metal-support coordination site being opened (state iii and state vii), deformation energies of both the adsorbates (green line) and the catalysts (red line) are large. Thus, it is seen in Fig. 3a (bottom) that the highly active intermediates and catalysts are held together by the strong metal-intermediate interactions, leading to the smooth reaction landscape shown in Fig. 3a (top). The opening of the metal-support coordination site with its strong distortion suggests a mechanism to intensify the strong metal-intermediate interaction, which stabilizes unstable intermediates (bent CO_2_* or OCOO*) and thus favors the activation. On the contrary, for states with the metal-support coordination site being closed (e.g., state iv and state viii), the deformation energy of the catalyst is released, which is accompanied by the formation of a stable intermediate (e.g. linear CO_2_*) as the product, while a weakened metal-adsorbate interaction facilitates the desorption of the product. For instance, the desorption of a linear CO_2_* (state iv) costs 0.11 eV less than the direct desorption of a bent CO_2_* (state iii), which is the consequence of Cu-O re-coordination through Cu migration to the nearby position with a barrier as low as 0.24 eV (Supplementary Fig. 10). The positions of Ce^3+^ in company with Cu migration are identified by using the Bader charges (Supplementary Fig. 11). The results show that the preferred locations are near the oxygen vacancy, which shall be beneficial to the energetics of Cu migration. In addition, our calculations show that, without hemilability to allow the participation of the closed state, the Cu center at the open state will bind too strongly the adsorbed species to hinder the evolution of the reaction (Supplementary Fig. 12). Hence, all these results demonstrate that the dynamic coordination of Cu SAC strengthens the bonding with the high-lying active intermediates with its open state to enhance the activation and weakens the bonding with the low-lying stable adsorbates with its closed state to facilitate the product desorption.

Compared to the substitute Cu_1_/CeO_2_ catalyst, the deformation energies of an extended Cu(111) surface are much mild (Fig. 3b). Despite that the deformation energies of the active intermediates are high as well, the metal-metal coordination does not invoke a similar mechanism for a comparably high deformation energy. Therefore, an optimal static catalyst, such as the extended metal surface with a structurally well-defined active center, requires a moderate bonding with the varying adsorbates to balance the activation and desorption as suggested by the Sabatier principle. Considering the dynamic nature of the metal-adsorbate coordination vs. the metal-support coordination in SACs^16,17^, this difference between the extended metal surfaces (or the nanoparticle catalysts with well-defined structures) and SACs shall provide a new insight into the unique reactivity of SACs. Certainly, it is worthy of note that the reconstruction of metal catalysts under the operando conditions may also create active sites where the local environments can be opened and closed reversibly driven by the dynamics of the adsorbates from the reactants to the intermediates and to the products, as the reaction proceeds^53^. In this way, the static optimum of the Sabatier volcano can be circumvented^54^.

### Dynamic electronic structure change driven by hemilability

**Fig. 4:**
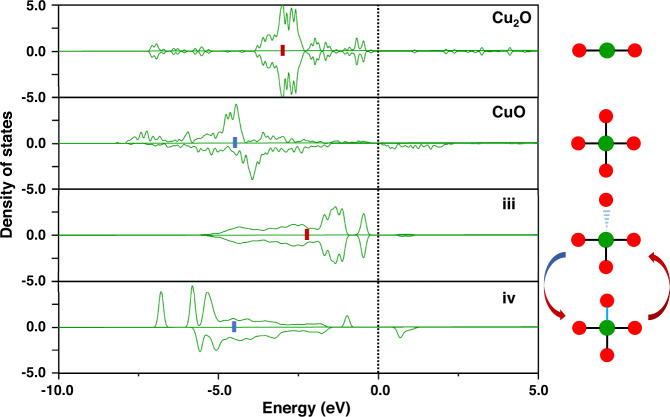
Density of states for the d-electrons of Cu with different metal-support coordinations. Cu(I) and Cu(II) in Cu_2_O and CuO bulks, respectively, are used as references to explore the effect of the local coordination environments on the d-electron structures of the Cu ion in the substituted Cu_1_/CeO_2_ catalyst. The dash line is the position of the Fermi-level. The blue or red bar indicate the positions of the d-band centers. The schemes to the right of the figure presents the respective local coordination environment of Cu. The green and red balls represent Cu and O, respectively. The metal-support coordinations change in company with the changes of the valence of Cu, i.e., open for Cu(I) and closed for Cu(II), as verified by comparing their spin polarizations to those in bulk Cu_2_^(I)^O and Cu^(II)^O. Source data are provided as a Source Data file.

For a better understanding of how the dynamic Cu-O metal-support coordination in the substituted Cu_1_/CeO_2_(111) tunes itself to adapt to the metal bondings with the adsorbates, the Cu d-band structures of state iii and state iv were calculated as illustrations to compare Cu in the opened and the closed metal-support coordinations for the substituted Cu_1_/CeO_2_(111). As shown in Fig. 4, the Cu d-electrons in state iii are closer to the Fermi level with a higher-lying d-band center, in comparison with those in state iv. When the number of the Cu-O coordination increases from three (state iii) to four (state iv), the Cu d-band center is stabilized, shifting away from the Fermi level. This is in consistency with the observation that the metal-adsorbate interaction in state iii is much stronger than that in state iv (see Supplementary Table 4 for the related adsorption bond strengths). Therefore, the opening and the closing of the dynamic metal-support coordination can significantly tailor the d-band center of the metal center, which makes an important contribution to intensify or weaken the metal-adsorbate bondings optimally.

The d-band centers for Cu in Cu_2_O and CuO bulks were also calculated for comparison (Fig. 4). The Cu(I) d-electrons in Cu_2_O bulk are higher-lying and spin-unpolarized, which provide a reference for state iii, whereas the Cu(II) d-electrons in CuO bulk are lower-lying and spin-polarized, which provide a reference for state iv. Bader charge analyses (Supplementary Table 1) also show that the change on the metal-support coordination leads to the change on the charge state of the metal, which is important to the change of the d-band center of the metal.

While a direct observation of the dynamic change for the opening or the closing of a metal-support coordination site is challenge, the above understanding on the relation between hemilability and the feature of the Cu ion, such as its coordination environment, charge state, d-band structure and binding ability, provides opportunities for observing and understanding hemilability in experiment. For CO adsorption and desorption on the Cu_1_/CeO_2_ catalyst, a recent operando XANES and EPR study has shown that the Cu(II) species changed to Cu(I) under CO with a decrease of the Cu–O coordination as observed by EXAFS spectra^55^, whereas the reverse was happening when the CO* was removed by the inert gases He or N_2_ gases only instead of by an oxidant as oxygen^55^. Such an observation could be understood by hemilability that CO adsorption opened the Cu–O coordination site to produce Cu(I) with a stronger binding ability, which, in turn, facilitated CO adsorption, while CO desorption led reversibly to the closing of the Cu–O coordination site along with the change of the oxidation state from Cu(I) to Cu(II). This hemilability was verified by the DFT calculations on both surfaces of the substituted Cu_1_/CeO_2_(111) (Supplementary Fig. 13) and the substituted Cu_1_/CeO_2_(110) (Supplementary Fig. 14). (More details about experiment and calculation can be found in Supplementary Note 2.1).

The key role of the dynamic coordination in achieving a high activity is further highlighted by comparing some other models of SACs. The first one is the adsorbed Cu_1_/CeO_2_(111) catalyst, which has been studied in a previous work^56^ similarly by means of the DFT + U method. Another one is the substituted Cu_1_/TiO_2_(110) catalyst. A detailed description of these two model catalysts are presented in Supplementary Method 1.2 and Supplementary Fig. 1b, c. We start by assuming that CO oxidations on these two catalysts follow the same mechanism as that on the substituted Cu_1_/CeO_2_(111) catalyst studied in the present work. Some other results on the substituted Au_1_/CeO_2_(111) and Zn_1_/CeO_2_(111) model catalysts can be found in Supplementary Note 2.2 and Supplementary Fig. 15.

**Fig. 5:**
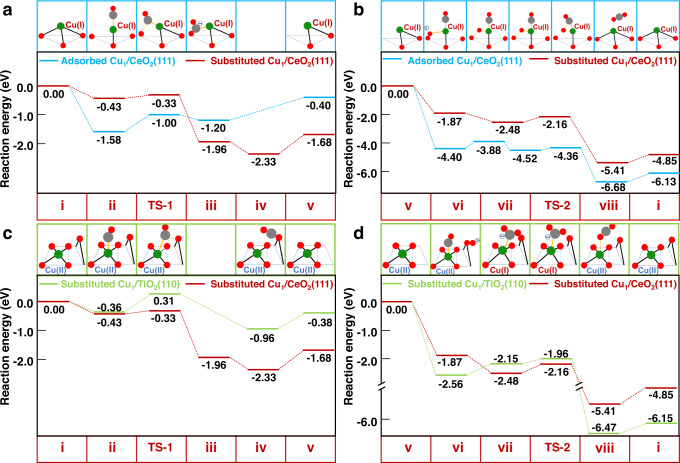
The comparisons of CO oxidation on other Cu SACs. **a** and **b** show the comparisons between the reaction energies on the substituted (red) and the adsorbed (blue) Cu_1_/CeO_2_(111) catalysts. The metal-support coordination in the adsorbed (blue) Cu_1_/CeO_2_(111) is very flexible, leading to too strong bindings with the adsorbates, which, in turn, results in the catalysts poisoning. **c** and **d** show the comparisons between the reaction energies on the substituted Cu_1_/CeO_2_(111) (red) and the substituted Cu_1_/TiO_2_(110) (green). The metal-support coordination in the substituted Cu_1_/TiO_2_(110) (green) is too rigid, leading to a higher effective barrier. The configuration schemes of the metal-adsorbate/metal-support coordinations are presented inside the corresponding small boxes. Please note the differences in the energy scales between **a** and **b**, as well as **c** and **d**.

As shown by the blue lines for energies and the corresponding boxes for structures in Fig. 5a, the metal-support coordination is very flexible in the adsorbed Cu_1_/CeO_2_(111), which adapts well to the CO adsorption. Hence, CO binds strongly on the adsorbed Cu_1_/CeO_2_(111) (−1.58 eV), followed by the CO oxidation reaction, which is endothermic by 0.38 eV. The as-formed CO_2_ also strongly binds on Cu(I) with a desorption energy of 0.80 eV. These two continuously endothermic steps add up to a very high energy cost of 1.18 eV, leading to an effective barrier of 0.76 eV, after adding a correction energy of −0.45 eV by considering the difference of the pre-exponential factors when actually comparing the rates of different processes. Similarly, as shown by blue lines and the corresponding boxes in Fig. 5b, CO and O_2_ bind strongly on the adsorbed Cu_1_/CeO_2_(111) (−4.40 eV), which are unable to couple favorably to form OCOO*, resulting in a 0.52 eV barrier for O_2_ dissociation and then a 0.16 eV barrier for CO_2_ formation. The results show that, for the metal-support coordination which is too flexible, it can strongly bind the adsorbates, which easily results in the catalyst poisoning. Therefore, the importance of having a proper metal-support coordination in favor of the product desorption is highlighted.

Now, we check the CO oxidation on the substituted Cu_1_/TiO_2_(110) catalyst. For the first half of the catalytic cycle, Fig. 5c shows a much high barrier of 0.67 eV than that on the substituted Cu_1_/CeO_2_(111) of 0.10 eV. The net reaction energy for the first half of the catalytic cycle on the former is −0.38 eV, while that on the latter is as low as −1.68 eV. This difference can be attributed to the fact that the eventually consumed lattice oxygen on the former is much more stable than that on the latter, as suggested by the corresponding oxygen vacancy formation energies of 2.89 eV and 1.59 eV, respectively, in the bridge (O^bri^) sites (Supplementary Table 5). It should be noted that the originally reactive lattice oxygen on the substituted Cu_1_/CeO_2_(111) was a 3-fold (O^3*f*^) coordinated lattice oxygen in state ii (see also Fig. 2 and Supplementary Fig. 1), which ended up at an O^bri^ as shown in state iv because of hemilability with the Cu migration. For the second half of the catalytic cycle as shown in Fig. 5d, despite to regenerate a more stable lattice oxygen, the step for CO* oxidized by O_2_* on the substituted Cu_1_/TiO_2_(110) has to surmount a higher effective barrier of 0.60 eV than that of 0.32 eV on the substituted Cu_1_/CeO_2_(111). On the substituted Cu_1_/CeO_2_(111), a Cu-O coordination site is opened to promote the formation of OCOO*. This mechanism is not working on the substituted Cu_1_/TiO_2_(110). Instead of opening a Cu-O coordination site, the Cu ion sticks out from the square planar to form a Cu(I)-bounded OCOO*. This displays the rigidity of the Cu-O coordination of the substituted Cu_1_/TiO_2_(110), as compared to that of the substituted Cu_1_/CeO_2_(111), in accordance with the respective oxygen vacancy formation energies (O^bri^: 3.39 eV v.s. O^3*f*^: 2.07 eV) as shown in Supplementary Table 5. Therefore, the comparison highlights the importance of having a proper metal-support coordination in favor of the reactant activation.

### Circumventing the scaling relationship by hemilability

**Fig. 6:**
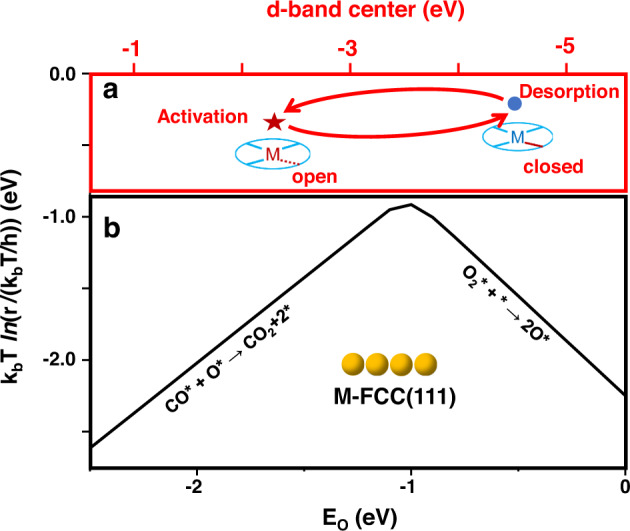
Comparison of two types of the activity maps for the catalytic CO oxidation. **a** On the substituted Cu_1_/CeO_2_(111) and (**b**) on the close-packed fcc(111) metal surfaces (Ni, Ru, Rh, Pd, Pt, Pd, Cu, Ag, Au). The 1-dimension activity volcano is shown in (**b**) with respect to the oxygen binding energy (E_O_, bottom horizontal axis), where the Sabatier volcano is applied to the close-packed fcc(111) metal surfaces using scaling relations reported in the literature work^57^. Each static fcc(111) surface corresponds to a static d-band center, yielding a specific E_O_. See more details in Supplementary Note 2.4 and Supplementary Fig. 16. On the contrary, the Sabatier volcano-plot cannot be simply applied to the substituted Cu_1_/CeO_2_(111) type of catalysts shown in (**a**), as hemilability can reversibly switch the metal center from one state to the other with a viable d-band center (top horizontal axis with the red star referring to the open state and the blue circle the closed state), which therefore can favor both the reactant activation and the product desorption simultaneously to circumvent the linear relationships. See more discussion in Supplementary Method 2.3. Source data are provided as a Source Data file.

The Sabatier volcano has been widely used as a powerful strategy for the rational design of an optimal catalyst^2^. This concept matches well with the widely used Langmuir model where the local coordination environment of the active center is well-defined and unmodified throughout the reaction pathway^53^. Therefore, all intermediates interact with a static electronic structure as characterized by a single value of the d-band center for a structurally well-defined active center, which, in turn, results in the coupling of their bonding strengths known as the scaling relationships^2,7,57^. Therefore, this guiding principle has also imposed limitations^25,43^, which people have been working hard to break^43^. It has been envisioned that a dynamic change of the catalyst structures can offer a way to circumvent the scaling relationships; however, its control at the micro level is nontrivial from the present tools available^43^. As enlightened here, SACs with hemilability can reversibly switch the metal center from one state to the other with a viable d-band center (top horizontal axis in Fig. 6a with the red star referring to the open state and the blue circle the closed state), which therefore can favor both the reactant activation and the product desorption optimally (See more discussion in Supplementary Note 2.3). In contrast, Fig. 6b shows the activity volcano for the catalytic CO oxidation on the well-defined close-packed fcc(111) metal surfaces constructed at 393.15 K under 1 atm CO and 1 atm O_2_ based on scaling relations reported in a literature work^57^. For clarity, only the 1-dimension cut parallel to oxygen binding energy (E_O_, bottom horizontal axis) at constant CO binding energy (E_CO_ = −0.6 eV) is shown, which crosses the top of the 2-dimension (E_O_ and E_CO_) activity volcano under the corresponding conditions (see more details in Supplementary Method 2.4 and Supplementary Fig. 16). Clearly, this type of Sabatier volcano with an static optimum (Fig. 6b) does not apply to SACs with hemilability (Fig. 6a). The latter provides a way to optimize the adsorption binding along the reaction pathway to achieve a higher activity as demonstrated in the substituted Cu_1_/CeO_2_(111) for CO oxidation.

### Distinguishing hemilability from other dynamics

To distinguish the concept of hemilability from the other dynamic behaviors in SACs, we have briefly summarized and compared some literature work that revealed dynamics of the active centers under reaction conditions. By comparing these cases as detailed in Supplementary Table 6, we have further classified the dynamics of the active centers into three types: (1) The dynamics of SACs caused by the hemilabile coordinations, which can be opened and closed reversibly in the catalytic cycles at the steady state as the reaction proceeds (cases 1–5)^31,58–61^. (2) The dynamic evolutions of SACs structure in response to different reaction conditions (cases 6–9), instead of the dynamic change in the catalytic cycles at a given condition^27,62–64^. (3) Other dynamic behaviors such as the phono-assisted dynamics of charge and oxidation state of the metal center (case 10)^39^. Even though type (2) has now been often discussed in literature, type (1) for the hemilabile coordinations has not yet been recognized. We provide here another example that can be better understood in terms of hemilability, where the hydrogenations of alkynes are catalyzed by a Pd SAC supported on mesoporous polymeric graphitic carbon nitride^65^. The DFT calculation results show that hemilability with the reversible opening and closing of the coordination site can favor the reactant activation and the product desorption simultaneously to achieve a higher activity (see Supplementary Note 2.5 and Supplementary Figs. 17–18 for details). More examples remain to be explored.

In summary, we have shown here that the reversible opening and closing of the metal-support coordination site significantly changes the electronic structure and the oxidation state of the Cu ion, which contributes to intensifying or weakening the metal-adsorbate bonding optimally to achieve a high CO oxidation activity as observed on the substituted Cu_1_/CeO_2_ catalyst. We relate this dynamic metal-support coordination to hemilability in SACs. As compared to the SAC with the hemilabile coordination, the model SACs with more labile or less labile coordinations are shown to suffer from the difficulties in either the product desorption or the reactant activation, respectively. While hemilability is one of the effective ways to regulate the reactivity of the catalyst in homogeneous catalysis, the introduction and application of this concept in heterogenous catalysis provides a new perspective for studying the dynamics of SACs, which shall be useful for the rational design of more sophisticated SACs.

## Methods

### DFT calculations

All periodic density functional theory (DFT) calculations were performed using Vienna ab initio simulation package (VASP)^66,67^. The core electrons were described by the projector augmented-wave (PAW) method. The kinetic energy cutoff for the plane wave basis sets of the valence electrons was set to be 400 eV. The surface Monkhorst–Pack meshes^68^ of 2 × 2 × 1 k-point sampling in the surface Brillouin zone were employed for all calculations. After the convergence criteria for optimizations were met, the largest remaining force on each atom was less than 0.02 eV Å^‒1^. The climbing image nudged-elastic band (CI-NEB) method^69^ was used to locate the transition state with a force tolerance of 0.03 eV/Å. The effective U values of 5.0 eV were used for both Ce 4f-orbitals, Cu 3d-orbitals, and 3.3 eV for Ti 3d-orbitals. For surface reactions, the contributions of the dispersive interactions were accounted for by using the DFT + D3 method with Becke-Jonson damping^70,71^. More details and discussions can be found in the Supplementary Methods. The XYZ coordinates for key reaction states can be found in the Supplementary Data 1.

### Kinetic Monte Carlo simulations

The mathematical foundation for KMC simulations was derived from the well-known chemical master equation (CME)^72^. Solving the CME analytically for general systems is unpractical, thus the rejection-free stochastic simulation algorithm (SSA) was developed to provide a numerical access^72^. A KMC simulation proceeds by generating a sequence of system configurations. At each configuration, the rates of all the possible elementary events are evaluated. Appropriately weighted by these different rates, one of the possible event is then executed randomly, leading to the next system configuration. This way, the KMC algorithm simulates stochastic processes described by a Poisson distribution, and a direct and unambiguous relationship to real time is established by advancing the clock

1 $$t=t-\frac{{{{{\mathrm{ln}}}}}({{{{{\rm{RN}}}}}})}{{R}_{tot}}$$

where RN is a uniform random number between 0 and 1, and *R*_tot_ is the sum of the rates of all the possible elementary events at current configuration. More details about KMC algorithm also can be found in the Supplementary Methods.

## Supplementary information

Supplementary Information

Description of Additional Supplementary Files

Supplementary Movie 1

Supplementary Movie 2

Supplementary Data 1

Source Code

## Data Availability

The data that support the findings of this study are available from the corresponding author upon reasonable request. Source data are provided with this paper.

## Source data

Source Data

## Code availability

A Python code for KMC simulation of CO oxidation on the substituted Cu_1_/CeO_2_(111) surface was provided as Supplemental Materials.
