# Supplementary material for: Dynamic evolution of the active center driven by hemilabile coordination in Cu/CeO2 single-atom catalyst
Source: Nat Commun. 2023 May 2;14:2512. doi: 10.1038/s41467-023-38307-w (PMC10154346; doi:10.1038/s41467-023-38307-w)
Supplement: Supplementary file 1 — Supplementary Information [file 41467_2023_38307_MOESM1_ESM.pdf]

Supporting Information for

Dynamic evolution of the active center driven by hemilabile coordination in Cu/CeO_2 single-atom catalyst

Zheng Chen[1], Zhangyun Liu[1], Xin Xu[1,2]*

[1]Collaborative Innovation Center of Chemistry for Energy Materials, Shanghai Key Laboratory of Molecular Catalysis and Innovative Materials, MOE Key Laboratory of Computational Physical Sciences, Department of Chemistry, Fudan University, Shanghai 200433, People's Republic of China.

[2]Hefei National Laboratory, Hefei 230088, P. R. China.

*E-mail: xxchem@fudan.edu.cn

ORCID Xin Xu: 0000-0002-5247-2937

Supplementary Methods……………………………………………………………3

1.1 Details for density functional theory calculations and ab initio molecular dynamic simulations………………………………………………………………3

1.2 Models for single Cu atom catalysts………………………………………5

1.3 Calculations for oxygen vacancy formation energy and deformation energy……7

1.4 Calculations for rate coefficients…………………………………………..8

1.5 Details for kinetic Monte Carlo simulations……………………………10

1.6 Calculations for kinetic reaction orders and apparent activation energy……...11

1.7 Stability of the substituted $Cu_1/CeO_2(111)$ under reaction conditions…………...13

Supplementary Notes……………………………………………………………14

2.1 Experimental evidence for the hemilability...……………………………14

2.2 OCOO* formation and dissociation on $Au_1/CeO_2(111)$ and $Zn_1/CeO_2(111)$ …....16

2.3 Scaling relations and the break-down of the scaling relations…………………17

2.4 Activity volcano for CO oxidation on close-packed fcc(111) metal surfaces…....19

2.5 Hydrogenations of alkynes on the Pd_1/mpg-C_3N_4 SAC……………………...21

Supplementary Figures…………………………………………………………...23

Supplementary Tables ……………………………………………………………41

Supplementary References………………………………………………………...47

Supplementary Methods

1.1 Details for density functional theory calculations and ab initio molecular dynamic simulations

All density functional theory (DFT) calculations were performed by using the Vienna ab initio simulation package (VASP).[1–3] The (1s) electrons in H, (2s, 2p) electrons in C/N/O, the (3d, 4s) electrons in Cu/Ti, (4d, 5s) electrons in Pd and (5s, 5p, 5d, 4f, 6s) electrons in Ce were treated as valence electrons, while the kinetic energy cutoff for the plane wave basis sets was set to be 400 eV. The surface Monkhorst–Pack meshes[4] of 2×2×1 k-point sampling in the surface Brillouin zone were employed in all calculations. For systems involving CeO_2(111), a 3×3 supercell of 12 atomic layers was used (Supplementary Fig. 1a and 1b), where the bottom five layers of atoms were fixed in their optimized bulk positions, whereas the top seven layers, as well as the adsorbates, were allowed to fully relax. For systems involving rutile (110), a 4×2 supercell of 12 atomic layers was used (Supplementary Fig. 1c), where the bottom five layers of atoms were fixed in their optimized bulk positions, whereas the top seven layers, as well as the adsorbates, were allowed to fully relax. After the convergence criterion for intermediate state optimizations has been met, the largest remaining force on each atom is less than 0.02 eV/Å. The climbing-image nudged elastic band (CI-NEB) method[5] was used to locate the transition state with a force tolerance of 0.03 eV/Å. The effective U values of 5.0 eV were used for both Ce 4f-orbitals, Cu 3d-orbitals,[6–8] and 3.3 eV for Ti 3d-orbitals.[9,10] Considering the dynamic changes of the Cu charges and the oxidation states, we have also tested the reasonability of using the effective U values of 5.0 eV for both Cu(I) and Cu(II). The results (Supplementary Table 2) showed that the effective U values of 5.0 eV, 6.0 eV and 7.0 eV result in almost the same adsorption energies of CO and CO_2 on Cu(II) and Cu(I), respectively. For surface reactions, the contributions of the dispersive interactions were accounted for by using the DFT + D3 method with Becke-Jonson damping.[11,12]

Ab initio molecular dynamics (AIMD) simulations based on Born−Oppenheimer approximation were also performed using VASP.[1–3] A time step of 2 fs was used.

Canonical (NVT) ensemble and Nosé−Hoover thermostats[13,14] were set to 800 K. Due to the short time scales, AIMD simulations can only sample very fast, low-energy-barrier events and may strongly depend on the initial configurations. We thus performed MD simulations at relatively high temperature to accelerate the sampling.

1.2 Models for single Cu atom catalysts

The $CeO_2(111)$ surface was chosen as the basement. To model the substituted $Cu_1/CeO_2(111)$ surfaces, one pair of Ce^{4+}–O^{2-} on the top layer was replaced by a Cu^{2+} ion, leading to the spontaneous formation of the first oxygen vacancy (V_O):[15]

$$[2Ce^{4+},O^{2-}] \rightarrow [Ce^{4+},Cu^{2+},V_O] + 1/2\ O_2 \quad \text{(S1)}$$

The as-formed (111) surface was depicted in Supplementary Fig. 1a. A previous work[16] has reported another configurations of the substituted $Cu_1/CeO_2(111)$ surface model, where Cu^{2+} is located on a 3O-coordinated site (diaphanous green ball in Supplementary Fig. 1a). However, by DFT optimizations, we found that the migration of Cu from the 3O-coordinated site to the near 4O-coordinated site results in a decrease in energy by 0.70 eV. Considering that the experiment[16] was performed at high temperature, the more stable model with 4O-coordinated Cu used in the present work would be more reasonable.

We also did the reasonability-check for the current Cu_1O_4 motif by comparing to the experimental characterization. In the previous experimental work,[16] a Cu_1O_3 active center was proposed based on the EXAFS fitting results that the more active catalyst possessed a main Cu−O shell ($R \approx 1.9$ Å, $CN \approx 3.3$). However, in a very recent work for Pt_1/CeO_2 single atom catalyst (SAC),[17] it has been shown that the EXAFS results could be well-fitted by different models (Pt_1O_3 or asymmetric Pt_1O_4). Employing a high-symmetry model led to a Pt–O shell with $CN \approx 3.5$, indicating a defect Pt_1O_3 motif. However, such a model was considered not reasonable for the air-exposed state, since it has been proposed that excess Pt-O bonds would form from ambient O_2, once there was a vacancy, resulting in a $CN > 4$.[18,19] Instead, improved fitting results were obtained from an asymmetric square-planar Pt_1O_4 geometry with three shorter Pt–O_S distances of 1.979 Å and one longer Pt–O_L distance of 2.051 Å. In the substituted Cu_1/CeO_2 active center model proposed in the present work, the DFT optimized structure showed that the Cu_1O_4 is also asymmetric with four Cu-O bonds as 1.927 Å, 1.921 Å, 1.964 Å and 1.977 Å, respectively. Analogy to the Pt_1/CeO_2, we would expect that both the Cu_1O_3

motif and the asymmetric Cu_1O_4 motif with shorter/longer Cu-O bonds could fit well with the EXAFS results. Considering the calculated stability, the Cu_1O_4 model employed in the present work would be more reasonable.

For the adsorbed $Cu_1/CeO_2(111)$ surfaces, a previous work[20] has shown that the Cu atom that binds to the oxygen-hollow site was the most stable. The corresponding optimized configuration was depicted in Supplementary Fig. 1b.

Similarly, to model the substituted $Cu_1/TiO_2(110)$ surfaces, one pair of Ti^{4+}–O^{2-} on the top layer was replaced by a Cu^{2+} ion, leading to the spontaneous formation of the first V_O:[15]

$$[2Ti^{4+},O^{2-}] \rightarrow [Ti^{4+},Cu^{2+},V_O] + 1/2\ O_2 \qquad (S2)$$

The as-formed (110) surface was depicted in Supplementary Fig. 1c. For a better comparison to the substituted $Cu_1/CeO_2(111)$ with Cu located in a square formed by four lattice oxygens, the spontaneous oxygen vacancy under Cu in the substituted $Cu_1/TiO_2(110)$ was chosen.

1.3 Calculations for oxygen vacancy formation energy and deformation energy

Besides the spontaneous Vo, the Vo formation energy is calculated as:

$$\Delta E(V_O) = E_t(Cu_1/M_xO_y) + 1/2\ E_t(O_2) - E_t(Cu_1/M_xO_{y-1}), \quad (S3)$$

where $E_t(Cu_1/M_xO_y)$ and $E_t(Cu_1/M_xO_{y-1})$ are the total energies of the optimized supercell before and after the Vo formation, respectively, while $E_t(O_2)$ is the total energy of a gas-phase O_2.

The deformation energy was defined as the energy difference between the conformation confined in combined system and the optimal conformation in the free state of the individual. In the present work, each state was decomposed into two components, namely the adsorbate (A) and the catalyst (C), respectively. Therefore, the adsorbate can be the reactant, the intermediate and the product along with the reaction. For any state A-C, the deformation energies of adsorbate $E_{def}(A)$ and the catalyst $E_{def}(C)$ were calculated, respectively, by

$$E_{def}(A) = E_t(A@A\text{-}C) - E_t(A_{opt}), \quad (S4)$$

$$E_{def}(C) = E_t(C@A\text{-}C) - E_t(C_{opt}), \quad (S5)$$

where $E_t(A@A\text{-}C)$ and $E_t(C@A\text{-}C)$ are the total energies of A and C with their conformations in A-C. $E_t(C_{opt})$ is the total energy of the catalyst in its relaxed conformation. $E_t(A_{opt})$ is the total energy of the adsorbate in its stable gas phase. Hence, A_{opt} for the adsorbed CO*, O_2*, CO_2* are the fully optimized gas phase molecules of CO, O_2 and CO_2, while A_{opt} for OCOO* is free CO and free O_2. Note that, the deformation energies of the transition states were not considered here, as it was hard to assign A_{opt} to the free reactant or the free product. Also the present choices of not using charged species, e.g. CO_2^- and $OOCO^-$, as the references to calculate the deformation energies reflect the fact that the structure deformation and the charge transfer are coupled to each other, both of which are induced by the strong covalent interactions between the adsorbates and the surfaces.

1.4 Calculations for rate coefficients

Collision theory and transition state theory were employed to calculate the rate coefficients of adsorption/desorption and reaction processes, respectively.[21] For adsorption processes, the rate coefficients are given by:[21]

$$k_{ads} = \frac{S_c^0(T)}{N_0\sqrt{2\pi m_A k_B T}}, \tag{S6}$$

where $S_c^0(T)$ is a coefficient related to the sticking coefficient, N_0 is the concentration of reaction sites per unit area on the surface, m_A is the molecular mass of species A, while k_B is the Boltzmann constant and T is the absolute temperature. For CO oxidation on metals, it was found that the sticking coefficient of CO was much higher than that of O_2.[22,23] An O_2 sticking coefficient about 0.1 was measured on $Zr_{0.1}Ce_{0.9}O_2$ surface at 400 K.[24] Therefore, in the present work, the O_2 sticking coefficient of 0.1 was employed, while the higher CO sticking coefficient was assumed to be 1.0.

Equation S6 implies that a mobile model would be used for describing the adsorption transition state.[21] On the other hand, the adsorbate is often bound rather strongly and can be considered as immobile.[21] Accordingly, the rate of desorption could be derived by:

$$k_{des} = \frac{S_c^0(T)P_A}{N_0\sqrt{2\pi m_A k_B T}}\frac{Q_A}{Q_{A*}}\exp\left(-\frac{\Delta E_{des}}{k_B T}\right), \tag{S7}$$

where Q_A and Q_{A*} are the partition functions of the gas phase A and the adsorbed A*, respectively. ΔE_{des} is the desorption energy. Since the changes of vibrational contributions before and after adsorption are usually small, only the rotational and translational contributions of the gas phase species A are considered in the present work. If A is considered as an ideal gas,[21] the transitional and rotational partition functions Q_A^{trans} and Q_A^{rot} were given by

$$Q_A^{trans} = \left(\frac{2\pi m_A k_B T}{h^2}\right)^{\frac{3}{2}} V, \tag{S8}$$

$$Q_A^{rot} = \frac{1}{\sigma^*}\frac{k_B T}{hB^{rot}} \text{ (Linear)}; \qquad Q_A^{rot} = \frac{1}{\sigma^*}\left(\frac{k_B T}{h}\right)^{3/2}\sqrt{\frac{\pi}{A^{rot}B^{rot}C^{rot}}} \text{ (nonlinear)}. \tag{S9}$$

Here $V = \frac{k_B T}{p_A}$ is the volume of the system, σ^* is the symmetry factor, A^{rot}, B^{rot}, C^{rot} are the rotational constants.

Transition state theory[25–27] is often used to calculate the rate coefficients for the heterogeneous catalytic reactions. For the reaction processes on lattice, the rate coefficients were calculated by:

$$k = \frac{k_B T}{h}\frac{Q_{TS}^{'}}{Q_{IS}}\exp\left(-\frac{E_a}{k_B T}\right), \tag{S10}$$

where E_a is the activation barrier, $Q_{TS}^{'}$ is the quasi-partition functions of the transition state, while Q_{IS} is the partition functions of the initial state. Despite the absolute values of vibrational partition functions were not very small, here again we assumed that the changes of vibrational partition functions before and after reaction were small, and thus could be ignored.

1.5 Kinetic Monte Carlo simulations

The general kinetic Monte Carlo (KMC) algorithm is also briefly introduced. The mathematical foundation for KMC simulations was derived from the well-known chemical master equation (CME).[28] Solving the CME analytically for general systems is unpractical, thus the rejection-free stochastic simulation algorithm (SSA) was developed to provide a numerical access.[28] In the SSA, the first step was to identify the rates of all the possible elementary events that could occur under a given configuration x $\{R_i(x)\}$. At each step, an event i was randomly selected with the probability described by

$$P_i = \frac{R_i(x)}{\sum_j R_j(x)} \cdot \qquad \text{(S11)}$$

After the event execution, the simulation time was subsequently updated by

$$\Delta t = -\frac{\ln(RN)}{\sum_j R_j(x)} \qquad \text{(S12)}$$

where RN is a uniform random number between 0 and 1. The whole procedure was repeated by simulating the currently most imminent event, thereby generating a stochastic trajectory that could be post-processed to yield activity.

1.6 Calculations for kinetic reaction orders and apparent activation energy

It is well-known that the PBE functional usually underestimated the activation barrier. For better comparison with the experimental results, more accurate functional is necessary, at least for the rate-determining step.[29] However, a higher accuracy usually means a lower efficiency. Thus, a combinational strategy, namely the extended ONIOM (XO)[30,31] method, was employed here, using the doubly hybrid functional XYG3[32–35] with basis set of 6-311+G(3df, 2p) as the high level (*H*) to correct the highest barrier, which is the dissociation of OCOO* with the original VASP calculations at the PBE level being the low level (*L*). In the XO calculations, the energies of the transition state (TS) and the initial state (IS) at the high level $E_{TS}(H)$ and $E_{IS}(H)$ was estimated by

$$E_{TS}(H) \approx E_{TS}^{cluster}(H) - E_{TS}^{cluster}(L) + E_{TS}(L), \quad \text{(S13)}$$

$$E_{IS}(H) \approx E_{IS}^{cluster}(H) - E_{IS}^{cluster}(L) + E_{IS}(L)\,. \quad \text{(S14)}$$

Therefore, the barrier at the high level could be calculated by equation S13 - equation S14:

$$E_a(H) = E_a^{cluster}(H) - E_a^{cluster}(L) + E_a(L)\,. \quad \text{(S15)}$$

The structures of [Cu-OCOO] cluster cut out from TS-2 and state vii from VASP PBE calculations, respectively, were used. The corrected barriers of OCOO* dissociation was calculated to be 0.58 eV, as summarized in Supplementary Table 3.

By using DFT calculation results (Fig. 2 in the main body) with the XO correction, collision theory and transition state theory (Supplementary Method 1.3) to obtain the rate constants, KMC simulations (Supplementary Method 1.5) were employed to estimate theoretical kinetic reaction orders and apparent activation energy, which can be compared to the experimental results,[16] directly. The free energy representations were obtained by the following equation

$$k = \frac{k_B T}{h}\exp\left(-\frac{G_a}{k_B T}\right). \quad \text{(S16)}$$

For a better comparison, the free energy barrier for the desorption step and the reaction step were both defined by equation S16. For example, Supplementary Fig. 9 shows the standard free energy landscape at 393.15 K. At this temperature, the kinetic reaction

orders of CO and O_2 were measured experimentally.[16]

With the rate constants being estimated under various conditions, the KMC simulations were employed to calculate the turnover frequency (TOF), and then to estimate the kinetic reaction orders and apparent activation energy for CO oxidation on the substituted $Cu_1/CeO_2(111)$. As shown in Supplementary Fig. 4, the simulated kinetic reaction orders of CO and O_2 are 0.71 and 0.81, respectively, which are in consistency with the experimental results of 0.95 and 0.89. Both the theoretical and the experimental kinetic orders agreed that the catalyst surface was not CO-saturated to impede the O_2 adsorption.[16] The simulated apparent activation energy is 0.59 eV (Supplementary Fig. 4c), which is also in good agreement with the experimental result of 0.48 eV.[16] All in all, the simulations of these key kinetic properties are in good agreement with the experiments, which support the proposed mechanism to provide a solid base for revealing usefulness of the hemilability concept in SACs.

1.7 Stability of the substituted $Cu_1/CeO_2(111)$ under reaction conditions

Besides the activity, it is important to examine the stability of the proposed active site under reaction conditions. In experiment, the high dispersion of copper was confirmed for both fresh and used samples, which means that no aggregation of copper occurred during reactions. As shown in Fig. 2 in the main text, the first lattice oxygen in state i can be easily consumed by CO* with a low barrier of 0.10 eV, while regeneration of the lattice oxygen requires to overcome a higher barrier of 0.32 eV. This indicates that states with a lattice oxygen vacancy should have a longer resident time. We have further examined the relative stabilities for configurations where single Cu atoms are located on different sites with/without adsorbed reactants (CO*/O_2*) as shown in Supplementary Fig. 2. The results show that the four oxygen coordinated single Cu atoms are the most stable in all cases (conformation I). With the induction of reactants (CO* or O_2*), the conformations with one Cu-O metal-support coordination opened are more stable as compared to case without any reactant (conformation II). However, when the single Cu atom migrates far away from the Ce defect, it become highly unstable for all cases with and without adsorbed reactants (conformations III and IV).

Supplementary Notes

2.1 Experimental evidence for the hemilability

In a recent experimental work,[36] the *operando* XANES and EPR studies have shown that Cu(II) was observed under air, whereas Cu(I) was observed under CO. The corresponding EXAFS spectra has shown that the Cu–O coordination number (CN) decreases from ~4 to ~2 in conjunction with the oxidation state of Cu from Cu(II) to Cu(I). Interestingly, when the inert gas He or N_2 was introduced to remove CO, a paramagnetic Cu(II) was observed again in the EPR and XANES studies with an increased Cu–O CN as observed by the EXAFS spectra. These results suggest that the adsorption of CO results in the Cu(I) species with a lower Cu-O CN (i.e. the open state), while the desorption of CO* by inert gases, instead of an oxidant, tends to close the coordination site of Cu and results in the Cu(II) species with a higher Cu-O CN (i.e. the closed state).

DFT calculations have been performed to provide atomic pictures of the hemilability observed by the experiment. The substituted models for the Cu SACs supported on both $CeO_2(110)$ and $CeO_2(111)$ surfaces have been chosen. As usual, the substituted single Cu^{2+} atom was introduced by replacing one pair of Ce^{4+}–O^{2-} with a pair of Cu^{2+}–V_O to maintain the charge balance. In accord with the experimental results that the CO desorption led to the oxidation of Cu(I) by Ce^{4+}, such that a Ce^{3+} should be produced in the surface, and an additional oxygen vacancy was also produced accordingly.

As shown in Supplementary Fig. 13a, the single Cu(II) ion on $CeO_2(110)$ was located in a square formed by four lattice oxygens, whose charge state was identified by the calculated Bader charge. When CO adsorbed onto the Cu site (Supplementary Fig. 13b and 13c), it preferred to induce the opening of the Cu-O coordination site along with the reduction of Cu(II) to Cu(I). The open state (as in Supplementary Fig. 13b) had a stronger CO* bonding as compared to that in the closed state (as in Supplementary Fig. 13c), because the former had a much higher d-band center as compared to 4 O-coordinated Cu(II) in the latter. This is in excellent agreement with the observations by

the *operando* XANES, EPR and EXAFS experiments.[36] Similarly, for the substituted $Cu_1/CeO_2(111)$ system (Supplementary Fig. 14), the adsorption of CO also induced the opening of the Cu-O coordination site and the reduction of the oxidation state of Cu.

It is also important to notice that the change of the oxidation state from Cu(I) to Cu(II) is in conjunction with Ce(IV) to Ce(III) of the support along with the V_O formation. While Cu(I)/Cu(II) have been widely used as dynamic redox systems through variable coordination of the hemilabile ligands in homogeneous catalysis,[37] the favorable combination of the metal center and the support for their redox properties shows a good example here in heterogeneous catalysis.

2.2 OCOO* formation and dissociation on $Au_1/CeO_2(111)$ and $Zn_1/CeO_2(111)$

To explore the importance of hemilability, we further compared the substituted $Au_1/CeO_2(111)$ and $Zn_1/CeO_2(111)$ to $Cu_1/CeO_2(111)$ and $Cu_1/TiO_2(110)$. Considering the highest barrier appears at the OCOO* dissociation step on the substituted $Cu_1/CeO_2(111)$, we focused on comparing the OCOO* formation and dissociation. As shown in Fig. 4d, the OCOO* formation and dissociation start on surface models with a Vo formed besides the spontaneous Vo, which are denoted as M_1/Surf-Vo (M_1=Au, Zn, Cu; Surf=$CeO_2(111)$, $TiO_2(110)$). As shown in Supplementary Fig. 15a and 15b, the metal centers of Au and Zn can also migrate to the nearby position from the most stable one with an energy cost of 0.02 eV and 0.26 eV respectively, which is obviously lower than that of 0.48 eV on $Cu_1/CeO_2(111)$-Vo (Supplementary Fig. 2a). It indicates that both metal-support coordinations for Au and Zn are more flexible than that for Cu on the substituted M_1/ $CeO_2(111)$.

As shown by the calculation results, the binding affinity for CO and O_2 co-adsorption on $Zn_1/CeO_2(111)$-Vo (-1.88 eV, Supplementary Fig. 15c) is almost the same as that on $Cu_1/CeO_2(111)$-Vo (-1.87 eV, Fig. 4d), while CO binds strongly to $Au_1/CeO_2(111)$-Vo (-2.54 eV, Supplementary Fig. 15c), being similar to that on $Cu_1/TiO_2(110)$-Vo (-2.56 eV, Fig. 4d). Eventually, OCOO* is formed exothermically with formation energies of -2.69 eV and -2.80 eV on $Au_1/CeO_2(111)$-Vo and $Zn_1/CeO_2(111)$-Vo (Supplementary Fig. 15c), respectively, both of which are more negative than the corresponding values on $Cu_1/CeO_2(111)$-Vo (-2.48 eV) and $Cu_1/TiO_2(110)$-Vo (-2.15 eV) (Fig. 4d). Therefore, in addition to the binding ability of the metal center, the flexibilities of $Au_1/CeO_2(111)$-Vo and $Zn_1/CeO_2(111)$-Vo to adapt OCOO* are also making important contributions to the more stable OCOO*, while the more rigid $Cu_1/TiO_2(110)$-Vo leads to the less stable OCOO* as discussed in the main text. The more stable OCOO* on $Au_1/CeO_2(111)$-Vo and $Zn_1/CeO_2(111)$-Vo results in higher dissociation barriers of 0.52 eV and 0.48 eV, as compared to that on $Cu_1/CeO_2(111)$-Vo and $Cu_1/TiO_2(110)$-Vo of 0.32 eV and 0.19 eV, respectively (Fig. 4d). Nevertheless, the formation of OCOO* on the rigid $Cu_1/TiO_2(110)$-Vo from adsorbed CO and O_2 is endothermic by 0.41 eV, leading to an effective barrier of 0.60 eV.

Therefore, the $Cu_1/CeO_2(111)$-Vo with hemilability, which is neither too rigid nor too flexible, makes the switch of the metal center neither too easy to result in a too stable OCOO*, nor too difficult to result in a too unstable OCOO*. It thus achieves a low effective barrier for OCOO* dissociation, which highlights the importance of having a hemilabile metal-support coordination.

2.3 Scaling relations and the break-down of the scaling relations

The d-band model has proven particularly useful in understanding bond formation and trends in reactivity among the transition metal surfaces. The d-band model of Hammer and Nørskov[39,40] was developed based on the narrow d-band limit of the Newns-Anderson model.[41,42] According to this model, the band of d-states for the metal surfaces participating in the adsorbate bindings can be approximated by an average d-state at the energy known as the d-band center, while a higher-lying d-band center with respect to the Fermi energy indicates the possibility of the formation of a larger number of empty anti-bonding states, thus enabling the formation of a stronger adsorbate binding. The d-band center theory utilizes the d-band center as a quantitative descriptor, which states that the variation in the adsorption energy from one transition metal surface to another correlates quantitatively with the shift of the d-band center from one transition metal surface to another. This provides the foundation of the scaling relations on the transition metal surfaces for static catalysts.[43]

Chemisorption can always be understood in terms of orbital interactions between the adsorbates and the surfaces. Therefore this picture of binding interactions in terms of d-band center is also valid qualitatively not only for transition metal surfaces, but also for transition metal oxides, peroxides, nitrides, and sulfide surfaces, etc., including SACs.[44,45] On the other hand, representing a surface well as a d-band center and using the d-band center as a quantitative descriptor as in the d-band center theory is not always valid. In fact, the break-down of this quantitative correlation provides a way to circumventing the Sabatier volcano such as for SACs with the hemilable metal-support coordination.

While the reactant activation prefers a strong binding to the surface, the product desorption prefers a weak binding. However, under the restriction of the scaling relations, the adsorption and desorption of the adsorbates cannot be independently optimized and the adsorption energies of various adsorbates are coupled together such that they are described by some common descriptors. The scaling relations and the resultant volcano plot, using d-band centers or adsorption strengths based on a common adsorbate, can efficiently guide the search of an optimal catalyst from a family of static

catalysts, which, however, also impose a restriction on the maximum rate that can be achieved for the given class of catalysts.

There are various strategies that have been proposed to achieve a higher activity by circumventing the Sabatier volcano. The most direct way is to modify the scaling relations. A strategy to decouple the binding energies of various adsorbates is to introduce multiple sites or phases by using alloys, oxide/metal interfaces, and promoters.[44,46,47] Different functional groups of the key intermediates can show specific preferences to different types of sites, thus interacting with varying electronic structures.

While decoupling of bindings of various adsorbates can be achieved in space, it also can be achieved in time as the reactions proceed. Thus, dynamics can offer a way to circumvent the scaling relationships. For instance, the reversible opening and closing of a hemilabile metal-support coordination site can significantly change the d-band structure of the metal center, enabling that different key intermediates are possible to interact with the corresponding d-band structures for optimal bindings. Therefore, unlike the transition metal bulks or SACs with a nearly fixed metal-support coordination environment, as usually emphasized, SACs with the hemilability decouples the binding energies of different adsorbates naturally. The hemilability, which allows to favor both the reactant activation and the product elimination simultaneously, provides a way that goes beyond the static optimum of the Sabatier volcano. Using dynamic ways to circumvent the scaling relationships have also been reported in other systems including fluxional small nanoparticles,[48] dynamic surface ligands.[49]

While decoupling of the bindings of different adsorbates provides more flexibilities in catalyst design, such strategies, including the hemilability, also give an expanded parameter space and require a much more elaborate search process to identify the optimal catalysts. Meanwhile, we attribute here the hemilability associated with Cu_1/CeO_2 as the reason behind the experimental fact that Cu is an outstanding dopant to favor the reactant activation and the product desorption simultaneously, thus resulting in a high activity for CO oxidation.[16]

2.4 Activity volcano of CO oxidation on close-packed fcc(111) metal surfaces

The activity volcano of catalytic CO oxidation on close-packed fcc(111) metal surfaces established in the present work is based on the scaling relations and the Sabatier rates, as reported in the previous work.[38] A brief illustration is presented here, while more details can be found in ref [38]. The mechanisms contain the following elementary reactions:

$$\mathrm{CO} + * \rightleftharpoons \mathrm{CO}^*, \tag{SR1}$$

$$\mathrm{O_2} + * \rightleftharpoons \mathrm{O_2^*}, \tag{SR2}$$

$$\mathrm{O_2^*} + * \rightleftharpoons 2\mathrm{O}^*, \tag{SR3}$$

$$\mathrm{O}^* + \mathrm{CO}^* \rightleftharpoons \mathrm{CO_2} + 2*. \tag{SR4}$$

Reactions (SR1) and (SR2) are considered to be fast so that they are in equilibrium. The transition state energies for SR3 (E_{TS3}) and SR4 (E_{TS4}) were found to scale linearly with the adsorption energies of CO (E_{CO}) and O (E_O), as E_{TS3}=1.39E_O+1.56 eV and E_{TS4}=0.70(E_O+E_{CO})+0.02 eV. Furthermore, the O_2 adsorption energy was found to scale with the O adsorption energy as E_{O2}=0.89E_O+0.17 eV.[38]

The Sabatier rate is the rate that the reaction will have if all coverages are optimum for each elementary reaction step. Such conditions may not be obtainable in reality, but the Sabatier rate provides an upper bound to the steady-state rate, which is exact under all reaction conditions. When the reverse reactions are neglected, the rates of reaction for (SR3) and (SR4) are maximized. The Sabatier rate is therefore calculated from the forward rates:

$$r_3^+ = k_3^+ \theta_{O_2} \theta_* , \tag{S17}$$

$$r_4^+ = k_3^+ \theta_O \theta_{CO} , \tag{S18}$$

where θ^*, θ_{O2}, θ_O, θ_{CO} are the coverages of free sites, O_2*, O* and CO*, respectively. In the previous study,[38] the optimum coverages for $\theta_*^{\max}$, $\theta_{CO}^{\max}$, $\theta_{O_2}^{\max}$ were found by first neglecting the coverage of atomic oxygen, while still assuming that (SR1) and (SR2) were in equilibrium. The Sabatier rate r for CO_2 formation is determined by the lowest Sabatier rate of either Reaction (SR3) or (SR4):

$$r = \min\{2k_3^+ \theta_{O_2}^{\max} \theta_*^{\max}, k_4^+ \theta_O^{\max} \theta_{CO}^{\max}\} , \tag{S19}$$

where the factor of 2 stems from the stoichiometric number for (SR3).

2.5 Hydrogenations of alkynes on the Pd_1/mpg-C_3N_4 SAC

It has been reported that a single-site palladium catalyst, where the Pd atoms were anchored into the cavities of mesoporous polymeric graphitic carbon nitride (Pd_1/mpg-C_3N_4), was prepared and applied to the hydrogenations of alkynes.[50] This experiment showed a high activity and a product selectivity in comparison with benchmark catalysts based on nanoparticles.[50] There were various standby sites for the cavities of mpg-C_3N_4, where the metal center could dynamically and reversibly adjust the metal-support coordination environment along the reaction pathway, in a way that was similar to that illustrated in Scheme 1b in the main text. This type of dynamics did not involve redox properties of the metal center and the support. It only involved the opening and the closing of the metal-support coordination to the second layer when Pd bonded to the C_2H_2/H_2 reactants and the C_2H_4 product, respectively. Hence, this example of hydrogenation of alkynes on Pd_1/mpg-C_3N_4 was further employed to demonstrate how hemilability could contribute to the high catalytic performance by means of DFT calculations.

Being the same as the previous work,[50] the C_3N_4 moieties were built in a graphitic form. After optimization on the bulk, a four-layered slab of the (0001) surface was built with a p(2x2) supercell. While the revised-PBE functional without dispersion correction was employed in the previous work,[50] here we employed the PBE function with the dispersive interactions being accounted for by using the DFT+ D3 method with Becke-Jonson damping[11,12] (see more computational details in supplementary methods S1.1). In consistency with the previous work,[50] the incorporation of atomic Pd could be either on the surface or in the subsurface with the latter being more stable. Thus, the subsurface Pd site was employed as the starting point, where a single Pd coordinated to both the first and the second layers (Supplementary Fig. 17). The acetylene molecule was used as a surrogate alkyne for the mechanism study as suggested by the previous work.[50] Considering that the barriers of alkyne hydrogenations on Pd_1/mpg-C_3N_4 were obviously lower than the ethylene desorption energy,[50] only the free energy landscape with intermediates was presented here.

As shown in Supplementary Fig. 17, C_2H_2 bonded strongly to Pd, with an

adsorption energy of -1.02 eV, which opened up the metal-support coordination and induced Pd to float on the surface. On this single Pd site with an opened metal-support coordination, the hydrogen molecule underwent a heterolytic dissociation, which was downhill by 0.09 eV, leaving one hydrogen bound to an N atom in the lattice and the other hydrogen to the Pd atom. On the contrary, it was found that the direct dissociation of a hydrogen molecule on the subsurface single Pd site with a closed metal-support coordination was uphill by 0.21 eV. These results suggested that opening the metal-support coordination by C_2H_2 adsorption could benefit the H_2 adsorption and activation. In the following steps, the adsorbed H atoms were transferred to the alkyne moiety one by one with obviously exothermic reaction energies, leading to C_2H_3 and C_2H_4, respectively. Finally, the as-formed C_2H_4 left the surface with a desorption energy of 0.97 eV, and the metal-support coordination to the second layer was reconstructed simultaneously upon C_2H_4 desorption. Thus the hemilability here was related to the Pd atom floating on the surface (i.e., open state) or bonded to the second layer (i.e., closed state). To illustrate the influence of the hemilability, comparison was made to the C_2H_4 desorption from the single Pd site on a monolayer C_3N_4 (denoted as Pd_1/mono-C_3N_4), where the possibility to reconstruct the metal-support coordination to the second layer was ruled out. The result show that the C_2H_4 desorption energy on Pd_1/mono-C_3N_4 was increased to 1.37 eV (Supplementary Fig. 18), close to that of 1.39 eV on the Pd(111) surface. It suggested that the hemilability could benefit the C_2H_4 product desorption, making contributions to the high selectivity by preventing over-hydrogenation and oligomerization.

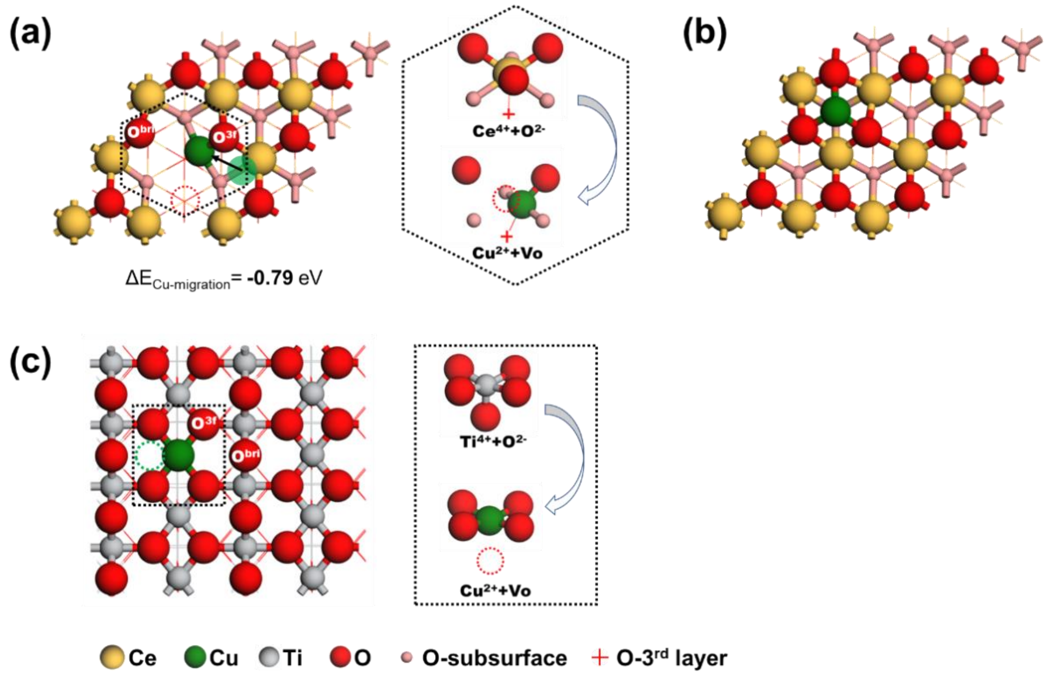

Supplementary Fig. 1 | Optimized structures for three model single Cu atom catalysts: (a) the substituted $Cu_1/CeO_2(111)$ catalyst, (b) the adsorbed $Cu_1/CeO_2(111)$ catalyst, and (c) the substituted $Cu_1/TiO_2(110)$ catalyst. O^{3f} and O^{bri} represent the 3-fold and the bridge sites of lattice oxygens, respectively. A dotted red cycle represents an oxygen vacancy (V_O) while the dotted green cycle in (**c**) represents the neighboring Cu site. A previous work[16] has reported another configurations of the substituted $Cu_1/CeO_2(111)$ surface model, where Cu^{2+} is located on a 3O-coordinated site (diaphanous green ball in (**a**)). However, we found that the migration of Cu from the 3O-coordinated site to a nearby 4O-coordinated site results in a decrease in energy by 0.70 eV.

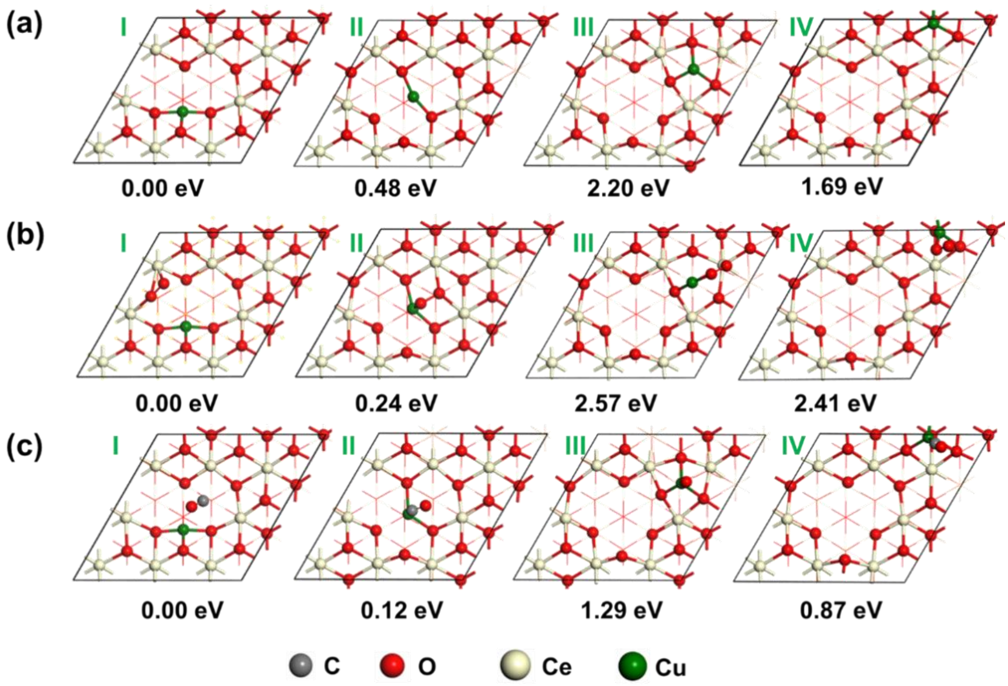

Supplementary Fig. 2 | Relative stabilities for single Cu atoms located on different coordination sites on the substituted $Cu_1/CeO_2(111)$ (a) without reactants, (b) with O_2*, and (c) with CO*, respectively. In conformation I, Cu is coordinated by four lattice oxygens forming a square planar close to the Ce defect. In conformation II, Cu is located on a symmetric position to that in conformation I. However, one of the coordinated lattice oxygens on the surface in conformation I is replaced by an oxygen vacancy. In conformation III, Cu is located on a 3O-coordinated site with one subsurface lattice oxygen and two surface lattice oxygens. Note that, the positions of three lattice oxygens are reconstructed slightly when they coordinated to Cu. In conformation IV, Cu is coordinated to a surface lattice oxygen only. All energies are referred to the corresponding structures of conformation I, which were used as the references.

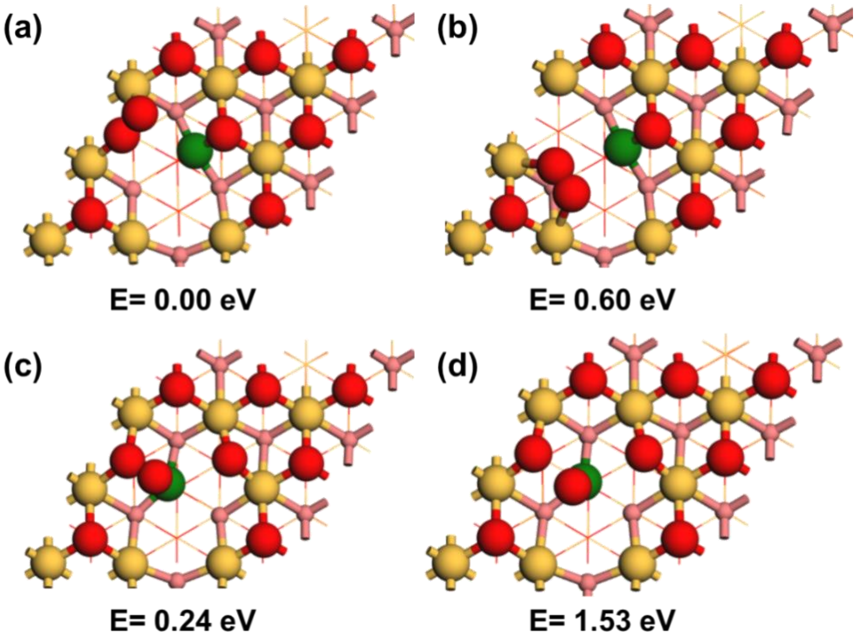

Supplementary Fig. 3 | Optimized structures and energies of intermediates for direct O_2* dissociation to 2O* on the substituted $Cu_1/CeO_2(111)$. (a) O_2* located on a single oxygen vacancy, (b) O_2* located between two oxygen vacancies, (c) O_2* located between an oxygen vacancy and a Cu, (d) O_2* dissociation over an oxygen vacancy and a Cu. The energy of O_2* located on a single oxygen vacancy is used as the reference energy. The results show that the energies of intermediates on either the direct dissociation pathway (b), or Cu-associated pathway (c and d) are much higher, as compared to the associative CO+O_2 pathway through OCOO* with an effective barrier of 0.32 eV discussed in the main text (**Fig. 1**).

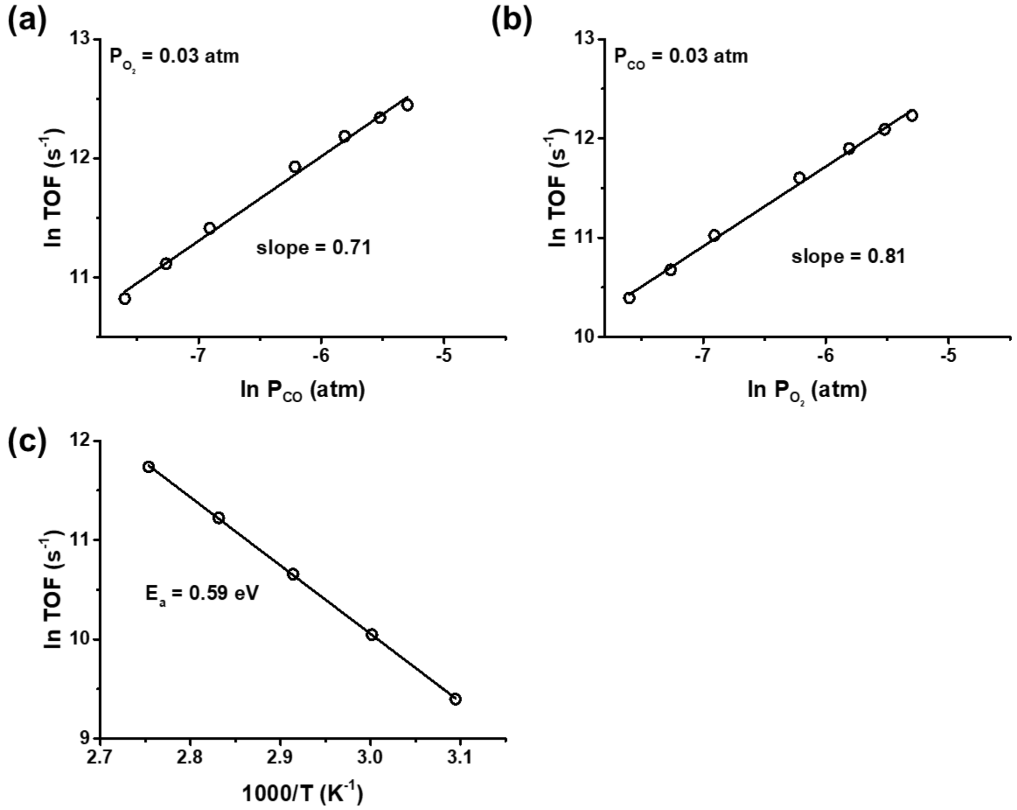

Supplementary Fig. 4 | Simulated kinetic reaction orders of (a) CO and (b) O_2 at 393.15 K for CO oxidation on the substituted $Cu_1/CeO_2(111)$. (c) Simulated apparent activation energy under P_{CO}=0.01 atm and P_{O2}=0.2 atm. The simulated kinetic orders of CO and O_2 are 0.71 and 0.81, respectively, which are in consistency with the corresponding experimental results of 0.95 and 0.89.[16] The simulated apparent activation energy is 0.59 eV, which is also in agreement with experiment result of 0.48 eV.[16]

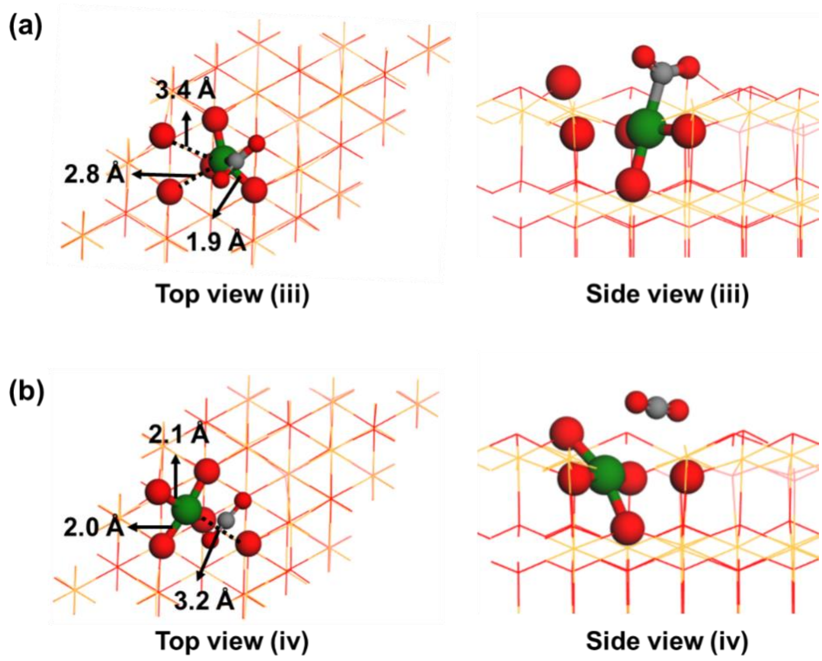

Supplementary Fig. 5 | Dynamic changes for the hemilabile metal-support coordinations along with the evolution of the adsorbate on the substituted $Cu_1/CeO_2(111)$. (a) In state **iii**, a hemilabile metal-support coordination is opened, which benefits the activation to form bended CO_2*. (b) In state **iv**, the hemilabile metal-support coordination is closed, which weakens the metal-adsorbate interaction and results in an inactive linear CO_2*.

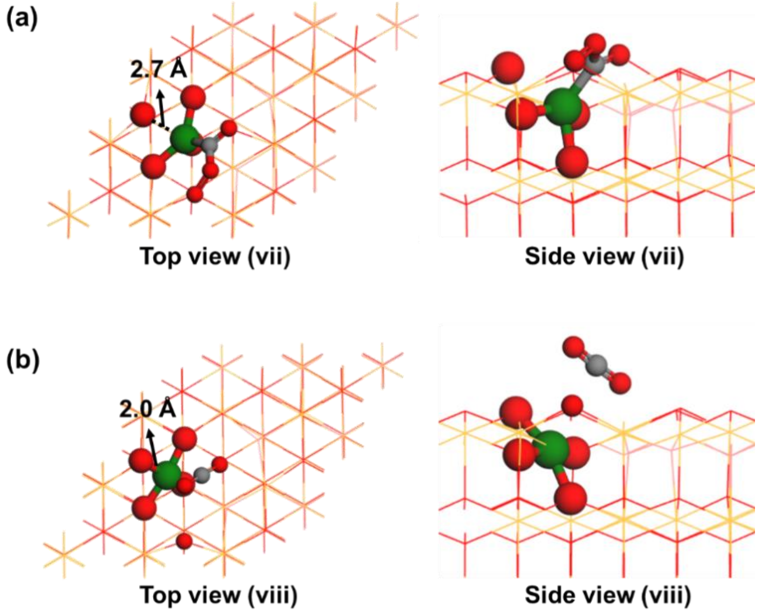

Supplementary Fig. 6 | Dynamic changes for the hemilabile metal-support coordinations along with the evolution of adsorbates on the substituted $Cu_1/CeO_2(111)$. (a) In state **vii**, a hemilabile metal-support coordination is opened, which benefits the formation of the active OCOO* species. (b) In state **viii**, the hemilabile metal-support coordination is closed after the dissociation of the active OCOO* species, which results in the formation of a linear CO_2* and regeneration of the lattice oxygen. See also Supplementary Fig. 5 and Supplementary Movies 1-2 for the ab initio molecular dynamic (AIMD) simulation results.

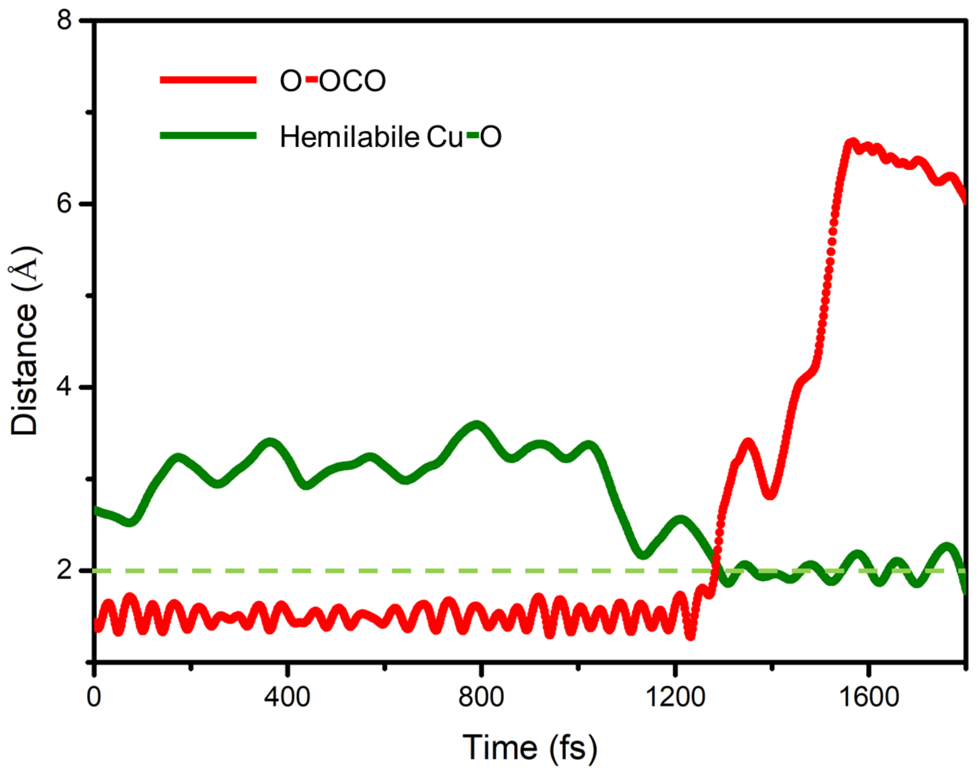

Supplementary Fig. 7 | AIMD simulations for the dissociation of OOCO* on the substituted $Cu_1/CeO_2(111)$. The red line refers to the O-O bond distance in OOCO*, while the green line refers to the distance of the hemilabile Cu-O coordination. The normal Cu-O metal-support bond distance is near 2 Å, which is presented as the dash green line. The simulations were performed at a relatively high temperature (800 K) to accelerate the sampling. Due to the short time scale, AIMD simulations can only sample the very fast events with low-energy-barriers. As the high temperature will result in fast desorption of CO* and CO_2* due to increasing entropy effects, the dissociation of OOCO* is simulated here as a representative.

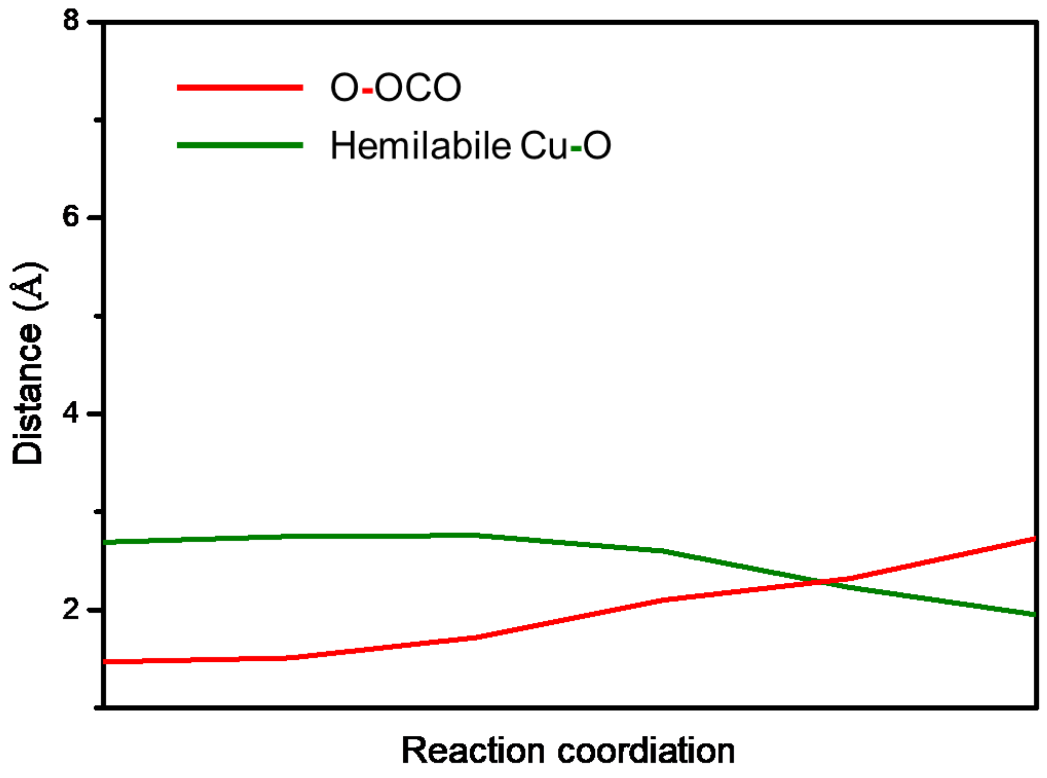

Supplementary Fig. 8 | Images of the nudged elastic band (NEB) method[5] for the dissociation of OOCO* on the substituted $Cu_1/CeO_2(111)$. Similar to the results of AIMD simulations, it is also shown that the re-coordination of the hemilabile Cu-O and the OCO-O* dissociation happen simultaneously.

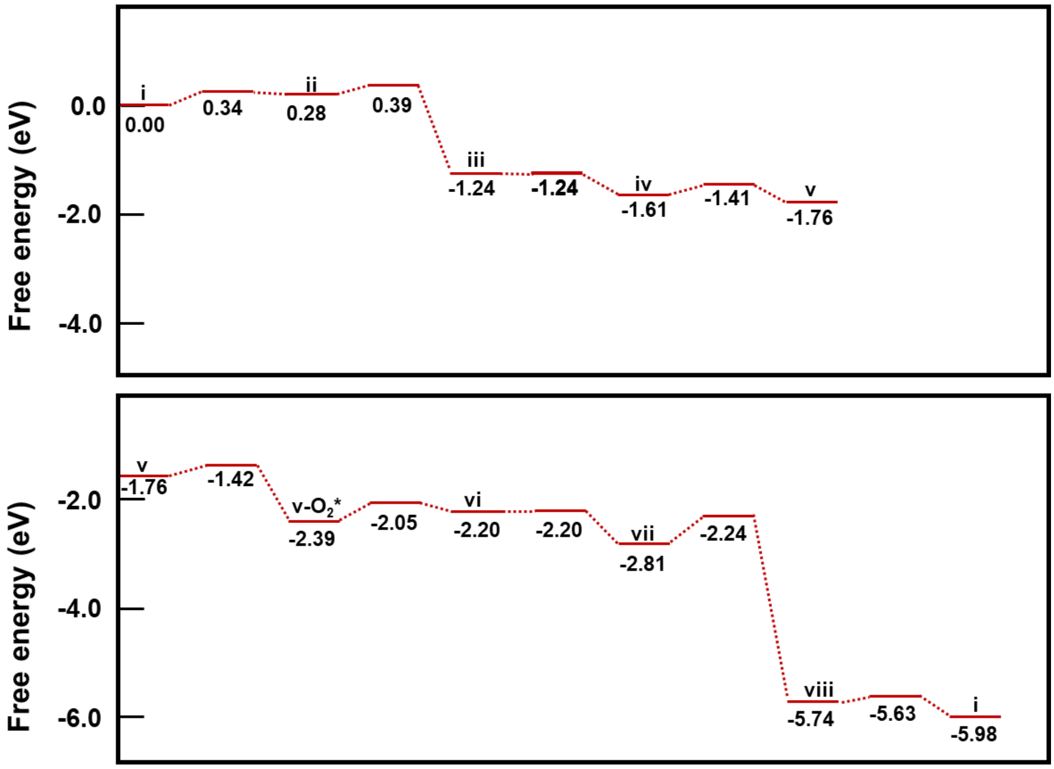

Supplementary Fig. 9 | Free energy landscape at 393.15 K for CO oxidation on the substituted $Cu_1/CeO_2(111)$. At this temperature, the kinetic reaction orders were measured experimentally. In the free energy landscape, the pre-exponential factors of all steps, including adsorption/desorption, are set as k_BT/h for comparison. Thus, although the CO_2* desorption step seems more endothermic (0.65 eV) in Figure 2 in the main body, it is actually a fast process due to its larger pre-exponential factor than that of surface reactions, which results in an effective barrier lower by 0.45 eV.

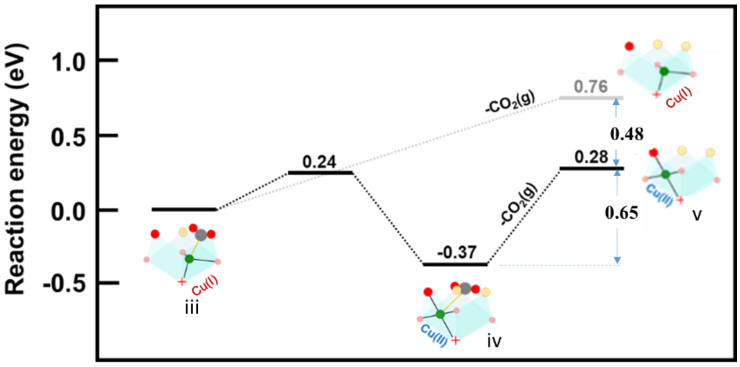

Supplementary Fig. 10 | Comparison of the two CO_2 desorption pathways from state **iii** on the substituted $Cu_1/CeO_2(111)$. Without invoking the hemiability, 0.76 eV is paid for the direct desorption of a bent CO_2^* (grey line). By invoking the hemiability, the Cu migration before CO_2^* desorption (black line) closes the Cu-O coordination in the first place, which results in a 0.11 eV lower CO_2^* desorption energy and stabilizes the Cu ion as Cu(II) by 0.48 eV. Clearly, the closing of the hemilabile Cu-O coordination strengthens the metal-support interaction and weakens the metal-intermediate interaction, which is beneficial to the CO_2^* desorption.

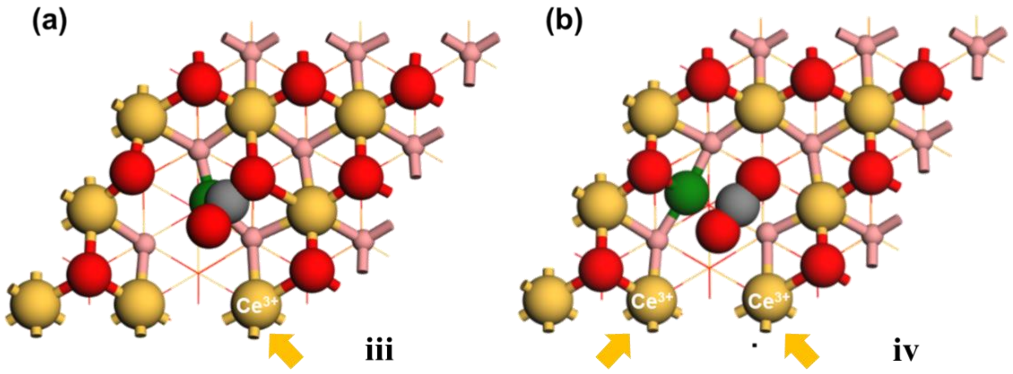

Supplementary Fig. 11 | The location of Ce^{3+} in states iii (**a**) and iv (**b**). The Ce^{3+} positions can be identified by the Bader charges. The results show that Ce^{3+} prefers to locate near the oxygen vacancy. This might be understood as Ce^{3+} with a larger volume prefers the more opened position such as the oxygen vacancy.

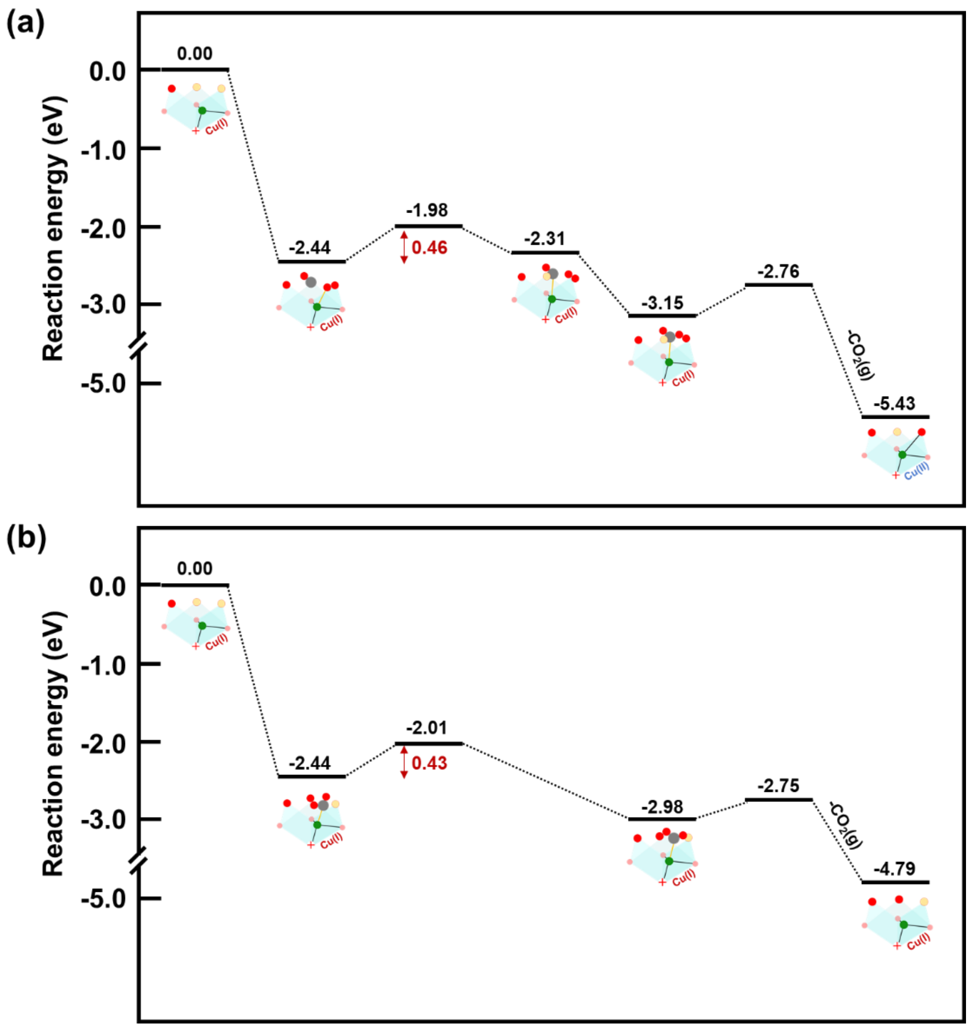

Supplementary Fig. 12 | The energy landscape for the OCOO* formation and its dissociation via constraint DFT calculations without invoking the hemilability to allow the migration of the Cu ion. The x and y coordination of the Cu ion was fixed at that of state **iii**, while the z direction was still allowed to relax. Since the Cu center was fixed on one side, the adsorptions of CO and O_2 on two oxygen vacancy sites were not equivalent, which led to two possible reaction pathways as shown in (**a**) and (**b**), respectively. In the case where the Cu migration was allowed (i.e., the hemilable mechanism), the slowest step showed a barrier of 0.32 eV (**Fig. 3** in the main text). However, without invoking the hemilability, pathway **(a)** showed that, O_2 strongly bound to the exposed Cu due to its high activity, which required a barrier of 0.46 eV for CO* to replace this O_2* for the formation of OCOO*; whereas pathway (**b**) showed that the exchange of CO* and O_2* led to a cis-OCOO* during optimization, which required a barrier of 0.43 eV to form OCOO*. Therefore, the results from constraint DFT calculations showed that, without invoking the hemilability, the exposing of the open state of Cu only was easy to bring in strongly bonded species (i.e. O_2* and cis-OCOO*), which resulted in higher barriers during the evolution of reaction than that with hemilability by exposing both closed and open states.

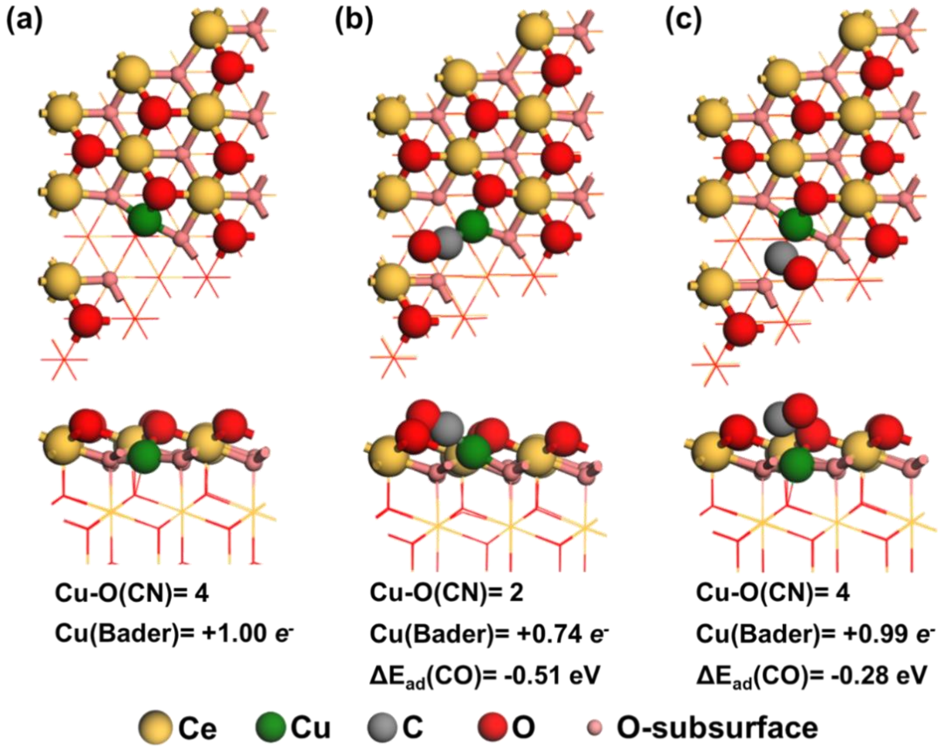

Supplementary Fig. 13 | The hemilability of the $Cu_1/CeO_2(111)$ surface caused by CO adsorption/desorption. The geometries, the Cu-O coordination numbers (CNs) and the Bader charge of Cu for the substituted $Cu_1/CeO_2(111)$ surface (a) without CO adsorption, (b) with CO adsorption in conjunction with a lower Cu-O CN (i.e., an opening state), (c) with CO adsorption in conjunction with a higher Cu-O CN (i.e., a closing state). As suggested by the adsorption energy $\Delta E_{ad}(CO)$, the adsorbed CO prefer the opening state over the closing state. And the desorption of CO* would result in an increased Cu-O CN back to 4 and the oxidation of Cu(I) to Cu(II), which is in consistency with the experimental observations by the *operando* EXAFS, XANES and EPR.

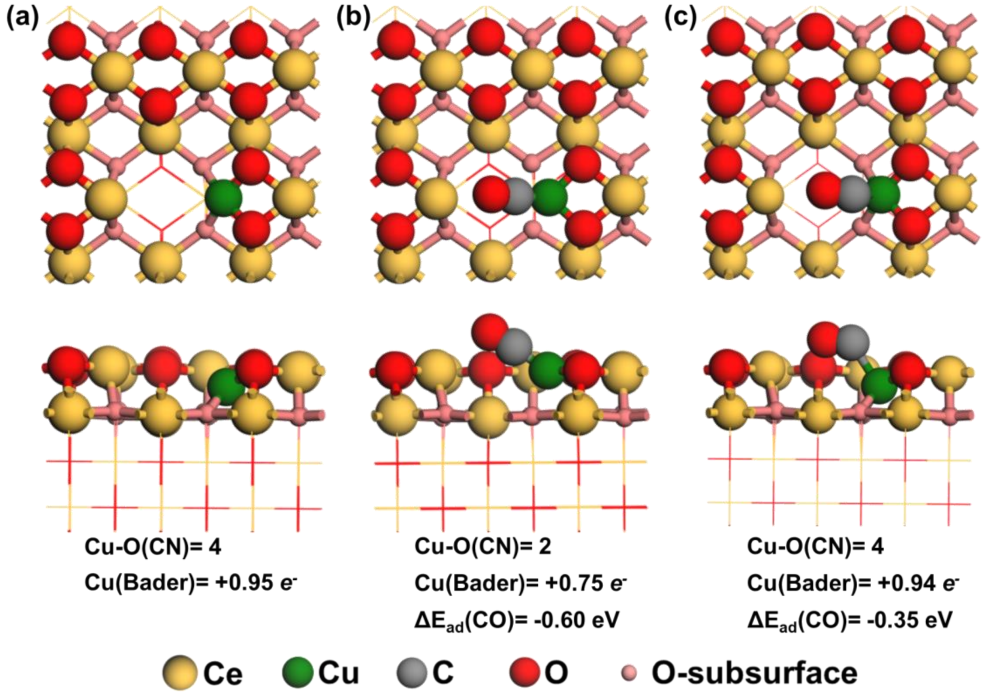

Supplementary Fig. 14 | The hemilability of the $Cu_1/CeO_2(110)$ surface caused by CO adsorption/desorption. The geometries, the Cu-O coordination numbers (CNs) and the Bader charge of Cu for the substituted $Cu_1/CeO_2(110)$ surface (a) without CO adsorption, (b) with CO adsorption in conjunction with a lower Cu-O CN (i.e., an opening state), (c) with CO adsorption in conjunction with a higher Cu-O CN (i.e., a closing state). As suggested by the adsorption energy ΔE_{ad}(CO), the adsorbed CO prefer the opening state over the closing state. And the desorption of CO* would result in the increased Cu-O CN and the oxidation of Cu(I) to Cu(II), which is in consistency with the experimental observations by the *operando* EXAFS, XANES and EPR.

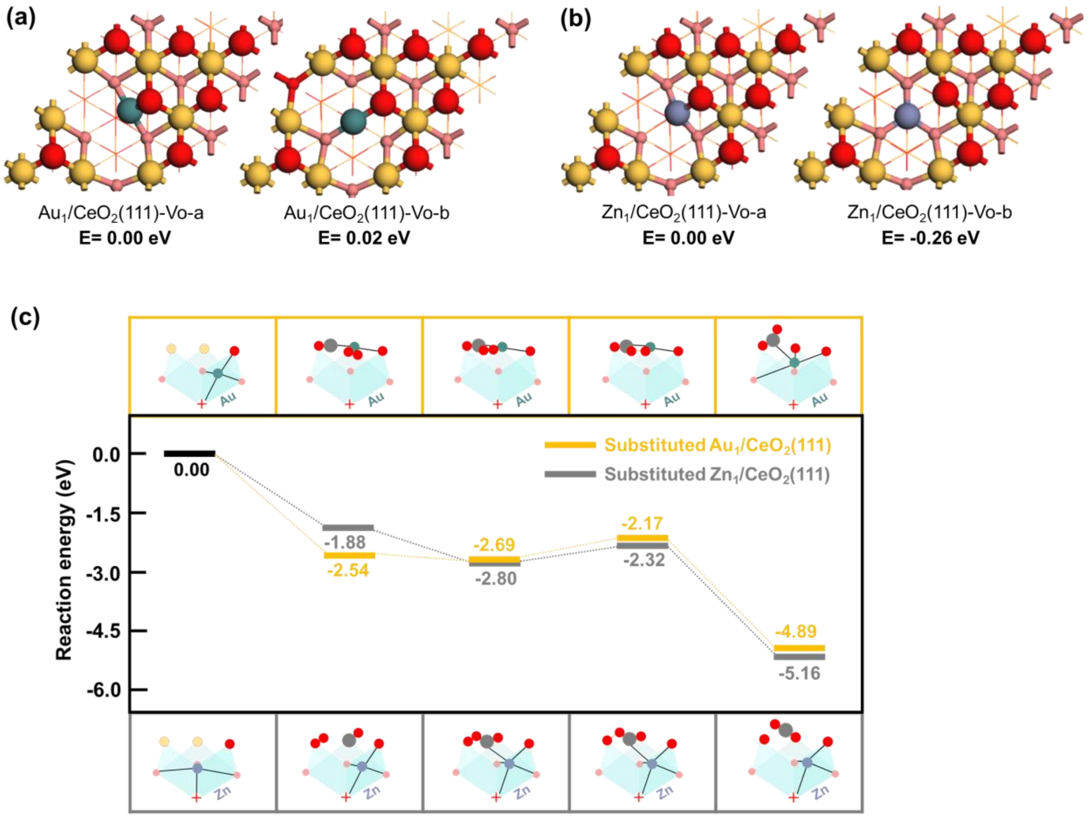

Supplementary Fig. 15 | OCOO* formation and dissociation on the substituted $Au_1/CeO_2(111)$ and $Zn_1/CeO_2(111)$. Two types of optimized structures for (**a**) $Au_1/CeO_2(111)$ and (**b**) $Zn_1/CeO_2(111)$, respectively, where one oxygen vacancy (Vo) exists besides the spontaneous Vo. The configuration with metal center located on the square formed by four O is chosen as the energy reference (E = 0.00 eV). (**c**) The corresponding reaction energy profiles for OCOO* formation and dissociation. For CO and O_2 co-adsorption on $Au_1/CeO_2(111)$, Au(II) changes from its square 4O-coordination to a Au(I) 3O-coordination; while for that on $Zn_1/CeO_2(111)$, Zn(II) migrates from its tetrahedral 4O-coordination to a square 4O-coordination.

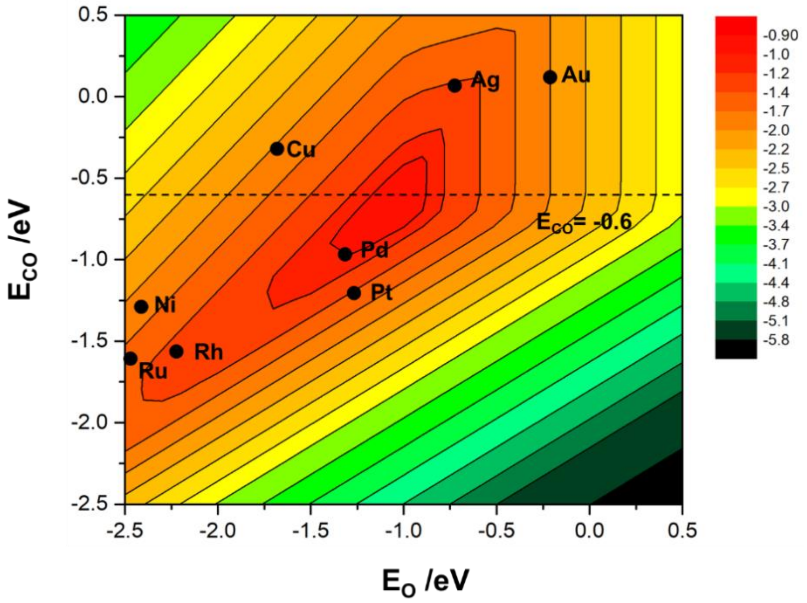

Supplementary Fig. 16 | Contour plot of the Sabatier activity $k_bT \ln(r/(k_bT/h))$ over close-packed metal surfaces as a function of E_{CO} and E_O at 393.15 K under 1 atm CO and 1 atm O_2 based on scaling relations reported in the previous work[38] (see details in Supplementary Notes 2.4).

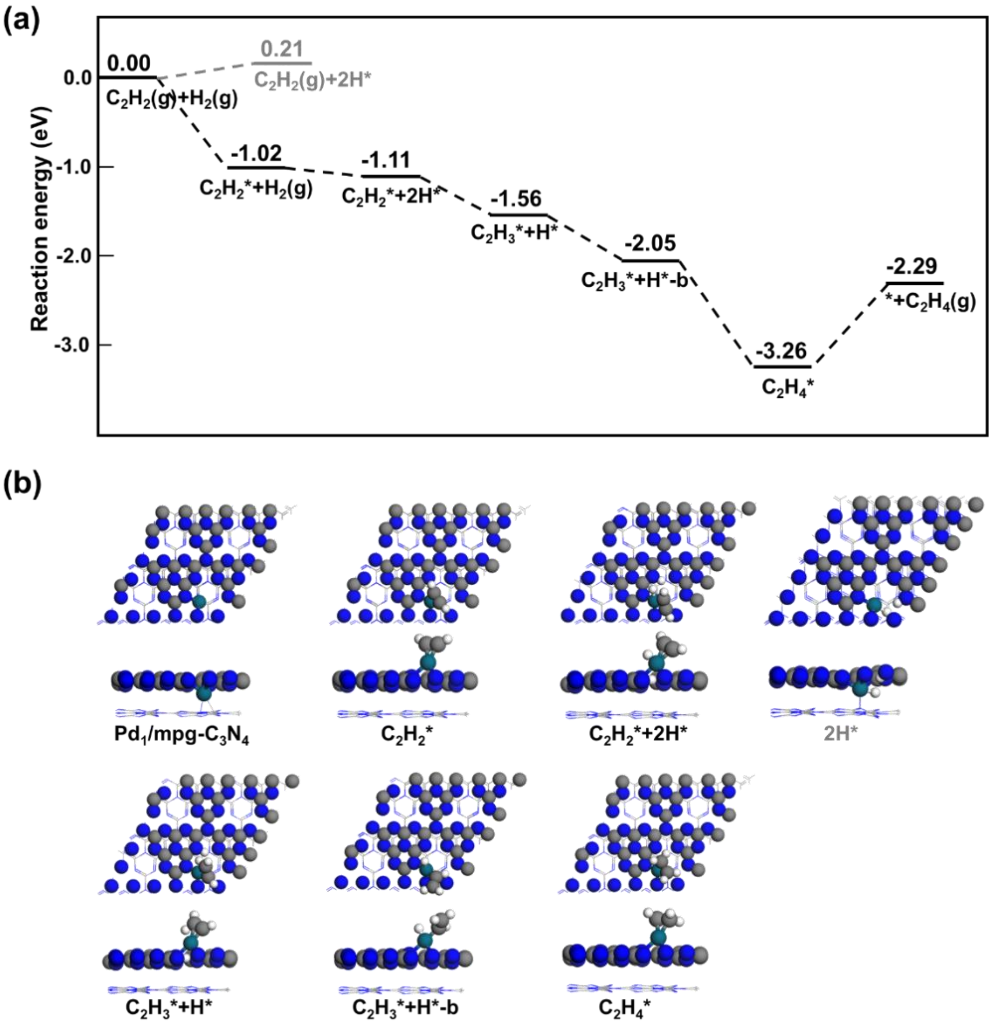

Supplementary Fig. 17 | Hydrogenations of alkynes on Pd_1/mpg-C_3N_4 SAC. (**a**) The reaction energy profile, and (**b**) the corresponding structures of intermediates in top view (top) and side view (bottom). H, C, N, and Pd are presented as while, grey, blue and dark green balls, respectively.

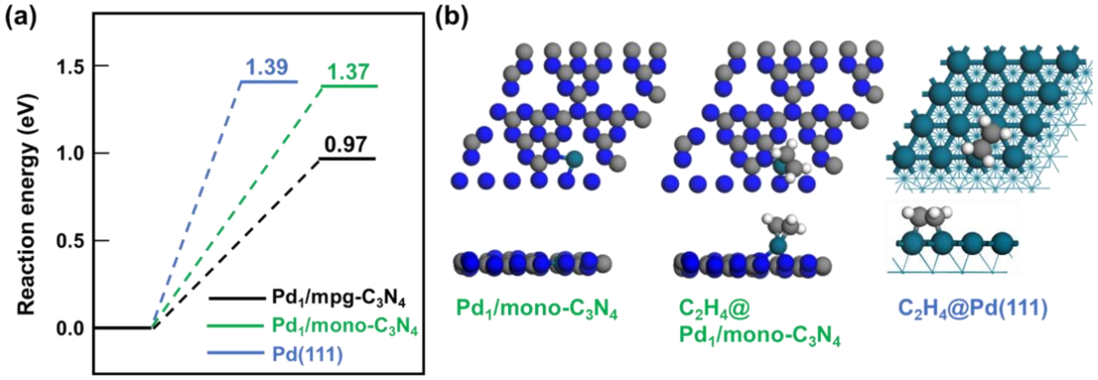

Supplementary Fig. 18 | The ethylene desorption on different types of catalysts. (**a**) The ethylene desorption energies from Pd_1/mpg-C_3N_4 (with hemilability), Pd_1/mono-C_3N_4 (without hemilability) and Pd(111) surfaces. (**b**) The optimized structures of Pd_1/mono-C_3N_4 and ethylene adsorption on Pd_1/mono-C_3N_4 and Pd(111). The structures for ethylene adsorption on Pd_1/mpg-C_3N_4 can be found in Supplementary Fig. 17. H, C, N, and Pd are presented as while, grey, blue and dark green balls, respectively.

Supplementary Table 1 | Bader charges of Cu in Cu_2O, CuO and the intermediate states on the substituted $Cu_1/CeO_2(111)$ SAC along the reaction.

State	Bader Charge	State	Bader Charge
Cu_2O	10.5		
CuO	10.0		
**i**	10.0	**v**	10.0
**ii**	10.0	**vi**	10.0
**TS-1**	10.0	**vii**	10.2
**iii**	10.2	**TS-2**	10.1
**iv**	10.0	**viii**	10.0

Supplementary Table 2 | The influence of effective U values, U_{eff}, on the calculated adsorption energies of CO and CO_2 on Cu(II) and Cu(I) in states **ii** and **iii**, respectively, on the substituted $Cu_1/CeO_2(111)$.

	U_{eff}=5.0	U_{eff}=6.0	U_{eff}=7.0
CO@Cu(II)/ii	-0.43	-0.43	-0.43
CO_2@Cu(I)/iii	-0.76	-0.70	-0.73

Supplementary Table 3 | Correcting barriers for the OCOO* dissociation on the substituted $Cu_1/CeO_2(111)$. Unit: eV

	PBE+U (*L*)/<br>PAW	PBE/<br>6-311+G(3df, 2p)	XYG3 (*H*)/<br>6-311+G(3df, 2p)
$E_a^{cluster}$	0.80	0.78	1.05
E_a	0.33	**-**	**0.58**

Supplementary Table 4 | The Cu-adsorbate bond strength in each state on the substituted $Cu_1/CeO_2(111)$ SAC along the reaction.

State	Cu-adsorbate bond strength (eV)	State	Cu-adsorbate bond strength (eV)
**i**	-*	**v**	-*
**ii**	-0.51	**vi**	-*
**iii**	-2.19	**vii**	-6.14
**iv**	-0.65	**viii**	-0.56

* No Cu-adsorbate bond.

Supplementary Table 5 | The oxygen vacancy formation energies on the substituted catalysts of $Cu_1/CeO_2(111)$ and $Cu_1/TiO_2(110)$. Unit: eV.

Oxygen position*	substituted $Cu_1/CeO_2(111)$	substituted $Cu_1/TiO_2(110)$
O^{bri}	1.59	—
O^{3f}	2.07	—
O^{bri}	—	2.89
O^{3f}	—	3.39

* See the label of oxygen in Supplementary Fig. 1

Supplementary Table 6 | Different types of dynamics for SACs under reaction conditions. Type (1): The dynamics of SACs, where the metal-support coordination can be opened and closed reversibly in the catalytic cycles in response to the metal-adsorbate coordination as the reaction proceeds from reactants to products (cases 1-5). This type of SAC dynamics is termed here as hemilability in SACs; Type (2): The SAC structures that dynamically evolve in response to the change of reaction conditions (e.g., oxidation, reduction, acidic and alkaline conditions as in cases 6-9), which then remain structurally well-defined at a given reaction condition; Type (3): Other dynamic behaviors (case 10).

Case	Different types of dynamics for SACs under reaction conditions	Reference
**1**	A four-coordinated nickel(II) confined in a zeolite, which can efficiently catalyze the selective hydrogenation of acetylene to ethylene. When the Ni bonds to an H after H_2 dissociation, one of the metal-support coordination is opened upon the Ni-H bond formation. The further adsorption of acetylene results in one more opened metal-support coordination. The re-coordination of the metal-support bonds happens one by one, along with the hydrogenation of acetylene to ethylene.	51
**2**	A four-coordinated Pt confined in the Y zeolite, which enables heterolytic dihydrogen activation and selective hydrogenations of α,β-unsaturated aldehydes to unsaturated alcohols. When the Pt bonds to an H after H_2 dissociation, one of the metal-support coordination is opened upon the Pt-H bond formation. According to the reported mechanism, their calculations demonstrated that a second opening of the metal-support coordination changed the orientation of Pt-H to facilitate the later hydrogenation. Upon hydrogenations, the opened metal-support coordinations are to be recoordinated, along with the product alcohol formation.	52
**3**	A heterogeneous SAC, where the Pd was anchored on an exfoliated graphitic carbon nitride (Pd-ECN), surpassing homogeneous systems for Suzuki coupling. With the adsorption of the reactants, the Pd-support coordinations were opened, which were closed with product desorption. Such an adaptive coordination environment within the macro-heterocycles of ECN facilitates each catalytic step.	53
**4**	A single gold atom in an oxidized-4-pyridinic cavity, designed for alkyne semi-hydrogenation. Two Au-N coordinations were opened after hydrogen dissociative adsorption on the Au atom, which were recoordinated after product desorption.	54
**5**	An SAC catalyst made of isolated single-atom Ru supported on mesoporous graphitic carbon nitride, which displayed an excellent hydrogenation and hydrodeoxygenation performance. Among three active site candidates, the	55

	calculation results have supported the one that bonds to the reactant vanillin most strongly with one opened Ru-C coordination.	
**6**	A uniform and well-defined Cu^{2+}-N_4 structure that exhibits comparable activity and superior durability in comparison to Pt/C. By combining operando X-ray absorption spectroscopy with theoretical calculations, the authors have identified the dynamic evolution of Cu-N_4 to Cu-N_3 under the ORR working conditions, which concurrently occurred with reduction of Cu^{2+} to Cu^{+}. It has been emphasized in this work that the dynamic structure evolution was mainly driven by the applied potential, rather than by the interaction with the reactants. The dynamics of the local coordination during the catalytic cycle at a given applied potential remains to be explored in detail.	56
**7**	Through a combination of in situ atomic-resolution microscopy and spectroscopy-based characterization supported by the first-principles calculations, it has been demonstrated that the isolated Pt species on TiO_2 surface can adopt a range of local coordination environments and oxidation states, which evolve in response to varied reaction conditions, such as oxidation, mild reduction and harsh reduction environments. Although the dynamic structure evolutions caused by the varied environmental conditions have been emphasized, whether there existed the dynamics of the local coordination in the catalytic cycle at a given environmental condition has not been mentioned and studied.	57
**8**	Dynamic evolution and the reversibility of single-atom Ni(II) active site in 1T-MoS2 electrocatalysts for hydrogen evolution under different reaction conditions. It has been evidenced that single-atom Ni(II) acted as the active species at the interface of Ni@1T-MoS2 in acidic conditions, while it underwent structure reconstruction in alkaline conditions to form a NiSxOy species that reversibly formed the catalytically active Ni^0 species under the applied potential.	58
**9**	Dynamically formed Au SAC. Under reaction conditions, Au single atoms could be dynamically created at the interface of small-sized Au nanoparticles on the ceria support, which accounted for the puzzling significant size effect in gold catalysis. This dynamic structure evolution from nanoparticles to single atoms has been related to the specified reaction conditions such as temperature, pressure, particle size, and the reducibility of the support.	59
**10**	Dynamics of charge and oxidation state of the Pt/CeO_2 SAC. A phonon-assisted dynamics has been demonstrated by combining density functional theory calculations and the first-principles molecular dynamic simulations on Pt single atoms deposited on the CeO_2 (100) surface.	60

Supplementary References

1. Kresse, G. & Hafner, J. *Ab initio* molecular dynamics for open-shell transition metals. *Phys. Rev. B* **48**, 13115–13118 (1993).
2. Kresse, G. & Furthmüller, J. Efficiency of ab-initio total energy calculations for metals and semiconductors using a plane-wave basis set. *Comput. Mater. Sci.* **6**, 15–50 (1996).
3. Kresse, G. & Furthmüller, J. Efficient iterative schemes for *ab initio* total-energy calculations using a plane-wave basis set. *Phys. Rev. B* **54**, 11169–11186 (1996).
4. Monkhorst, H. J. & Pack, J. D. Special points for Brillouin-zone integrations. *Phys. Rev. B* **13**, 5188–5192 (1976).
5. Henkelman, G., Uberuaga, B. P. & Jónsson, H. A climbing image nudged elastic band method for finding saddle points and minimum energy paths. *J. Chem. Phys.* **113**, 9901–9904 (2000).
6. Cui, L. *et al.* First-principles investigation of transition metal atom M (M = Cu, Ag, Au) adsorption on CeO2(110). *Phys. Chem. Chem. Phys.* **14**, 1923 (2012).
7. Nolan, M. Enhanced oxygen vacancy formation in ceria (111) and (110) surfaces doped with divalent cations. *J. Mater. Chem.* **21**, 9160 (2011).
8. Wang, Y. *et al.* Single-atomic cu with multiple oxygen vacancies on ceria for electrocatalytic CO_2 reduction to CH_4. *ACS Catal.* **8**, 7113–7119 (2018).
9. Cococcioni, M. & de Gironcoli, S. Linear response approach to the calculation of the effective interaction parameters in the LDA+U method. *Phys. Rev. B* **71**,

035105 (2005).

10. Selcuk, S. & Selloni, A. Facet-dependent trapping and dynamics of excess electrons at anatase TiO_2 surfaces and aqueous interfaces. *Nat. Mater.* **15**, 1107–1112 (2016).
11. Grimme, S., Antony, J., Ehrlich, S. & Krieg, H. A consistent and accurate *ab initio* parametrization of density functional dispersion correction (DFT-D) for the 94 elements H-Pu. *J. Chem. Phys.* **132**, 154104 (2010).
12. Grimme, S., Ehrlich, S. & Goerigk, L. Effect of the damping function in dispersion corrected density functional theory. *J. Comput. Chem.* **32**, 1456–1465 (2011).
13. Nosé, S. A unified formulation of the constant temperature molecular dynamics methods. *J. Chem. Phys.* **81**, 511–519 (1984).
14. Hoover, W. G. Canonical dynamics: Equilibrium phase-space distributions. *Phys. Rev. A* **31**, 1695–1697 (1985).
15. Paier, J., Penschke, C. & Sauer, J. Oxygen defects and surface chemistry of ceria: quantum chemical studies compared to experiment. *Chem. Rev.* **113**, 3949–3985 (2013).
16. Yu, W.-Z. *et al.* Construction of active site in a sintered copper-ceria nanorod catalyst. *J. Am. Chem. Soc.* **141**, 17548–17557 (2019).
17. Jiang, D. *et al.* Tailoring the local environment of platinum in single-atom Pt_1/CeO_2 catalysts for robust low-temperature CO oxidation. *Angew. Chem. Int. Ed.* **60**, 26054–26062 (2021).
18. Kunwar, D. *et al.* Stabilizing high metal loadings of thermally stable platinum single atoms on an industrial catalyst support. *ACS Catal.* **9**, 3978–3990 (2019).

19. Maurer, F. *et al.* Tracking the formation, fate and consequence for catalytic activity of Pt single sites on CeO_2. *Nat. Catal.* **3**, 824–833 (2020).

20. Qin, L., Cui, Y.-Q., Deng, T.-L., Wei, F.-H. & Zhang, X.-F. Highly stable and active Cu_1/CeO_2 single-atom catalyst for CO oxidation: A DFT Study. *ChemPhysChem* **19**, 3346–3349 (2018).

21. Chorkendorff, I. & Niemantsverdriet, J. W. *Concepts of Modern Catalysis and Kinetics*. (Wiley-VCH Verlag GmbH & Co. KGaA, Weinheim, 2003).

22. Yeo, Y. Y., Vattuone, L. & King, D. A. Calorimetric heats for CO and oxygen adsorption and for the catalytic CO oxidation reaction on Pt{111}. *J. Chem. Phys.* **106**, 392–401 (1997).

23. Bu, Y., Er, S., Niemantsverdriet, J. W. (Hans) & Fredriksson, H. O. A. Preferential oxidation of CO in H_2 on Cu and Cu/CeOx catalysts studied by in situ UV–Vis and mass spectrometry and DFT. *J. Catal.* **357**, 176–187 (2018).

24. Kolekar, S. K., Dubey, A., Date, K. S., Datar, S. & Gopinath, C. S. An attempt to correlate surface physics with chemical properties: molecular beam and Kelvin probe investigations of $Ce_{1-x}Zr_xO_2$ thin films. *Phys. Chem. Chem. Phys.* **18**, 27594–27602 (2016).

25. Evans, M. G. & Polanyi, M. Some applications of the transition state method to the calculation of reaction velocities, especially in solution. *Trans. Faraday Soc.* **31**, 875 (1935).

26. Eyring, H. The activated complex in chemical reactions. *J. Chem. Phys.* **3**, 107–115 (1935).

27. Laidler, K. J., Glasstone, S. & Eyring, H. Application of the theory of absolute reaction rates to heterogeneous processes II. Chemical reactions on surfaces. *J. Chem. Phys.* **8**, 667–676 (1940).

28. Gillespie, D. T. Stochastic simulation of chemical kinetics. *Annu. Rev. Phys. Chem.* **58**, 35–55 (2007).

29. Campbell, C. T. The degree of rate control: A powerful tool for catalysis research. *ACS Catal.* **7**, 2770–2779 (2017).

30. Guo, W., Wu, A. & Xu, X. XO: An extended ONIOM method for accurate and efficient geometry optimization of large molecules. *Chem. Phys. Lett.* **498**, 203–208 (2010).

31. Guo, W., Wu, A., Zhang, I. Y. & Xu, X. XO: An extended ONIOM method for accurate and efficient modeling of large systems. *J. Comput. Chem.* **33**, 2142–2160 (2012).

32. Zhang, Y., Xu, X. & Goddard, W. A. Doubly hybrid density functional for accurate descriptions of nonbond interactions, thermochemistry, and thermochemical kinetics. *Proc. Natl. Acad. Sci.* **106**, 4963–4968 (2009).

33. Wang, Y., Li, Y., Chen, J., Zhang, I. Y. & Xu, X. Doubly hybrid functionals close to chemical accuracy for both finite and extended systems: Implementation and test of XYG3 and XYGJ-OS. *JACS Au* **1**, 543–549 (2021).

34. Su, N. Q. & Xu, X. The XYG3 type of doubly hybrid density functionals: XYG3 type of doubly hybrid density functionals. *Wiley Interdiscip. Rev. Comput. Mol. Sci.* **6**, 721–747 (2016).

35. Su, N. Q. & Xu, X. Development of new density functional approximations. *Annu. Rev. Phys. Chem.* **68**, 155–182 (2017).

36. Kang, L. *et al.* Adsorption and activation of molecular oxygen over atomic copper(I/II) site on ceria. *Nat. Commun.* **11**, 4008 (2020).

37. Kaim, W., Beyer, K., Filippou, V. & Záliš, S. Charge and spin coupling in copper compounds with hemilabile noninnocent ligands – Ambivalence in three dimensions. *Coord. Chem. Rev.* **355**, 173–179 (2018).

38. Falsig, H. *et al.* Trends in the catalytic CO oxidation activity of nanoparticles. *Angew. Chem.* **120**, 4913–4917 (2008).

39. Hammer, B. & Nørskov, J. K. Why gold is the noblest of all metals. *Nature* **376**, 238–240 (1995).

40. Nørskov, J. K., Abild-Pedersen, F., Studt, F. & Bligaard, T. Density functional theory in surface chemistry and catalysis. *Proc. Natl. Acad. Sci.* **108**, 937–943 (2011).

41. Newns, D. M. Self-consistent model of hydrogen chemisorption. *Phys. Rev.* **178**, 1123–1135 (1969).

42. Anderson, P. W. Localized magnetic states in metals. *Phys. Rev.* **124**, 41–53 (1961).

43. Abild-Pedersen, F. *et al.* Scaling properties of adsorption energies for hydrogen-containing molecules on transition-metal surfaces. *Phys. Rev. Lett.* **99**, 016105 (2007).

44. Greeley, J. Theoretical heterogeneous catalysis: scaling relationships and computational catalyst design. *Annu. Rev. Chem. Biomol. Eng.* **7**, 605–635 (2016).

45. Liu, X., Jiao, Y., Zheng, Y., Jaroniec, M. & Qiao, S.-Z. Building up a picture of the electrocatalytic nitrogen reduction activity of transition metal single-atom catalysts. *J. Am. Chem. Soc.* **141**, 9664–9672 (2019).

46. Peterson, A. A. & Nørskov, J. K. Activity descriptors for CO_2 electroreduction to methane on transition-metal catalysts. *J. Phys. Chem. Lett.* **3**, 251–258 (2012).

47. Pérez-Ramírez, J. & López, N. Strategies to break linear scaling relationships. *Nat. Catal.* (2019) doi:10.1038/s41929-019-0376-6.

48. Zandkarimi, B. & Alexandrova, A. N. Dynamics of subnanometer pt clusters can break the scaling relationships in catalysis. *J. Phys. Chem. Lett.* **10**, 460–467 (2019).

49. Ortuño, M. A. & López, N. Creating cavities at palladium–phosphine interfaces for enhanced selectivity in heterogeneous biomass conversion. *ACS Catal.* **8**, 6138–6145 (2018).

50. Vilé, G. *et al.* A stable single-site palladium catalyst for hydrogenations. *Angew. Chem. Int. Ed.* **54**, 11265–11269 (2015).

51. Chai, Y. *et al.* Acetylene-selective hydrogenation catalyzed by cationic nickel confined in zeolite. *J. Am. Chem. Soc.* **141**, 9920–9927 (2019).

52. Deng, X. *et al.* Zeolite-encaged isolated platinum ions enable heterolytic dihydrogen activation and selective hydrogenations. *J. Am. Chem. Soc.* **143**, 20898–20906 (2021).

53. Chen, Z. *et al.* A heterogeneous single-atom palladium catalyst surpassing homogeneous systems for Suzuki coupling. *Nat. Nanotechnol.* **13**, 702–707 (2018).

54. Lin, R. *et al.* Design of single gold atoms on nitrogen-doped carbon for molecular

recognition in alkyne semi-hydrogenation. *Angew. Chem. Int. Ed.* **58**, 504–509 (2019).

55. Tian, S. *et al.* Temperature-controlled selectivity of hydrogenation and hydrodeoxygenation in the conversion of biomass molecule by the Ru_1/mpg-C_3N_4 catalyst. *J. Am. Chem. Soc.* **140**, 11161–11164 (2018).

56. Yang, J. *et al.* Dynamic behavior of single-atom catalysts in electrocatalysis: identification of cu-n_3 as an active site for the oxygen reduction reaction. *J. Am. Chem. Soc.* **143**, 14530–14539 (2021).

57. DeRita, L. *et al.* Structural evolution of atomically dispersed Pt catalysts dictates reactivity. *Nat. Mater.* **18**, 746–751 (2019).

58. Pattengale, B. *et al.* Dynamic evolution and reversibility of single-atom Ni(II) active site in 1T-MoS2 electrocatalysts for hydrogen evolution. *Nat. Commun.* **11**, 4114 (2020).

59. Liu, J.-C., Wang, Y.-G. & Li, J. Toward rational design of oxide-supported single-atom catalysts: atomic dispersion of gold on ceria. *J. Am. Chem. Soc.* **139**, 6190–6199 (2017).

60. Daelman, N., Capdevila-Cortada, M. & López, N. Dynamic charge and oxidation state of Pt/CeO_2 single-atom catalysts. *Nat. Mater.* **18**, 1215–1221 (2019).
