# Supplementary material for: Dynamic evolution of the active center driven by hemilabile coordination in Cu/CeO2 single-atom catalyst
Source: Nat Commun. 2023 May 2;14:2512. doi: 10.1038/s41467-023-38307-w (PMC10154346; doi:10.1038/s41467-023-38307-w)
Supplement: Supplementary file 2 — Description of Additional Supplementary Files [file 41467_2023_38307_MOESM2_ESM.pdf]

Description of Additional Supplementary Files

File Name: Supplementary Movie 1
Description: The top view of OCOO* dissociation in MD simulations.

File Name: Supplementary Movie 2
Description: The side view of OCOO* dissociation in MD simulations.

File Name: Supplementary Data 1
Description: XYZ coordinates for key reaction states for CO oxidation on various single atom catalysts (Fig. 2, Fig. 5 and Supplementary Fig. 15), and hydrogenations of alkynes on Pd1/mpg-C3N4 single atom catalyst (Supplementary Fig. 17-18).
